# Quantification of Skeletal Muscle at the First Lumbar Level for Prognosis in Amyotrophic Lateral Sclerosis

**DOI:** 10.1002/jcsm.13827

**Published:** 2025-06-05

**Authors:** Yujia Cao, Baoyu Yuan, Xiuyu Jiang, Chunming Xie, Di Wu, Zhijun Zhang

**Affiliations:** ^1^ Department of Neurology, Affiliated Zhongda Hospital, School of Medicine, Institution of Neuropsychiatry, Key Laboratory of Developmental Genes and Human Disease Southeast University Nanjing Jiangsu China; ^2^ Jiangsu Key Laboratory of Molecular and Functional Imaging, Department of Radiology, Zhongda Hospital, School of Medicine Southeast University Nanjing Jiangsu China

**Keywords:** amyotrophic lateral sclerosis, first lumbar level, nomogram, prognosis, skeletal muscle

## Abstract

**Background:**

Skeletal muscle parameters at the first lumbar vertebra (L1) level on computed tomography (CT) are common indicators for muscle mass. However, their relationship with the severity and prognosis of amyotrophic lateral sclerosis (ALS) patients remains unclear.

**Methods:**

This cohort study included ALS patients who underwent chest CT scans between January 2018 and January 2022 and healthy controls (HCs) matched for gender and age. Overall survival (OS) was determined from the date of chest CT to death, tracheal intubation or 1 January 2024. Using ImageJ software, skeletal muscle area and density (L1 SMA/SMD), skeletal muscle index (L1 SMI), paraspinal muscle area and density (L1 PMA/PMD) and subcutaneous fat area and density (L1 SFA/SFD) at L1 were quantified. The relationships between the quantified muscle parameters and both King's clinical stages and the Revised ALS Functional Rating Scale (ALSFRS‐R) were analysed. The Cox proportional hazard model was used to evaluate the hazard ratio (HR) of skeletal muscle parameters as risk factors for outcome events, and to construct a nomogram.

**Results:**

Muscle parameters in ALS patients (*n* = 102; 36.27% female; mean age, 60.85 ± 10.58 years) were significantly lower compared with HCs (*p* < 0.001). L1 SMD (*p* = 0.047) and L1 PMD (*p* = 0.003) both differed significantly across the King's clinical stages. ALSFRS‐R scores correlated with L1 SMA (*r* = 0.35, *p* < 0.001), L1 SMI (*r* = 0.34, *p* < 0.001), L1 PMA (*r* = 0.27, *p* = 0.007) and L1 PMD (*r* = 0.27, *p* = 0.007). Multivariate Cox regression analysis revealed that L1 SMA (HR = 0.96, 95% confidence interval [CI] = 0.94–0.98, *p* = 0.001), L1 SMD (HR = 0.92, 95% CI = 0.88–0.96, *p* < 0.001) and L1 PMA (HR = 1.06, 95% CI = 1.01–1.11, *p* = 0.022) significantly influenced ALS survival, with area under the curves (AUCs) of 0.687 and 0.851 for 1‐ and 3‐year OS prediction. The consistency index (C‐index) for the nomogram was 0.72 (95% CI = 0.641–0.793).

**Conclusions:**

Skeletal muscle parameters at L1 level on CT are significantly associated with clinical severity and prognosis in ALS.

**Trial Registration:**

Chinese Clinical Trial Registration Center: ChiCTR230078702

## 1 Introduction

Amyotrophic lateral sclerosis (ALS), a rapidly progressive neurodegenerative disease, is characterized by progressive dyskinesia and muscle atrophy that leads to respiratory failure and death within 3–5 years [1]. Understanding the factors that influence disease progression and prognosis in ALS is crucial for developing effective management strategies. Weight loss and low body mass index (BMI) at diagnosis are known adverse prognostic factors for ALS, emphasizing the importance of muscle mass for disease outcomes [2, 3]. Muscle denervation, decreased caloric intake and metabolic disturbances contribute to the wasting of skeletal muscle during disease progression [4]. In ALS, skeletal muscle dysfunction arises not only from the degeneration of lower motor neurons but also from intrinsic muscle abnormalities. Moreover, skeletal muscle dysfunction can contribute to the degeneration of the neuromuscular junction (NMJ) and motor neurons [5]. Mutations in genes associated with ALS (e.g., *SOD1* and *FUS*) downregulate key transcription factors and contractile proteins in satellite cells, impairing their ability to differentiate and limiting repair capacity [6, 7]. Muscle degeneration is exacerbated by intracellular dysfunctions, such as mitochondrial malfunction and disturbances in protein homeostasis [8]. Moreover, muscle cells from ALS patients release pro‐inflammatory signals and toxic metabolites, which adversely affect the NMJ and motor neurons [9]. Therefore, skeletal muscle plays a pivotal role in the progression of ALS.

Skeletal muscle mass assessment commonly relies on bioelectrical impedance analysis (BIA), dual‐energy X‐ray absorptiometry (DXA), computed tomography (CT) and magnetic resonance imaging (MRI). BIA provides an assessment of lean body mass and fat mass, whereas DXA estimates fat‐free mass and fat mass. However, neither BIA nor DXA provides a spatially resolved distribution of muscle and adipose tissue. Conversely, CT and MRI provide high‐resolution anatomical images that enable accurate visualization and quantification of specific muscle groups [10]. There is growing interest in utilizing CT to assess whole‐body or localized skeletal muscle status at a defined anatomical level, with the potential to predict disease prognosis. CT offers precise differentiation between muscle and fat, facilitating rapid scanning times [11]. In addition, it provides detailed information regarding myosteatosis and muscle atrophy [12]. The value of quantitative skeletal muscle parameters from chest CT as prognostic indicators has been validated in various respiratory diseases. Several studies have reported that low skeletal muscle quantitative parameters on chest CT, such as skeletal muscle index (SMI), skeletal muscle density (SMD), skeletal muscle area (SMA) and paravertebral muscle area (PMA), are associated with poor prognosis in respiratory‐related acute and chronic diseases, including lung cancer [13], chronic obstructive pulmonary disease [14] and severe pneumonia [15]. In chest CT, the first lumbar vertebra (L1) level is considered optimal for evaluating skeletal muscle mass. A large cohort study suggests that SMI at the 11th thoracic to L1 levels, particularly at L1, is strongly associated with whole‐body muscle mass [16]. Severe respiratory failure and pulmonary infections are the leading causes of mortality in ALS [17]. This is closely linked to the progressive weakening of L1 core muscles, including the psoas major, rectus abdominis and paraspinal muscles, which function as expiratory muscles. Their deterioration results in ineffective coughing, upper airway secretion retention and an increased risk of lower respiratory tract infections [18]. Studies have linked core muscle involvement in ALS to poor prognosis. Cecilia Marini et al.'s PET‐CT study found that ALS patients who died had reduced lumbar muscle volume and elevated FDG uptake compared with survivors [19]. Electrophysiologic studies have shown that denervation of the lower thoracic paraspinal [20] and rectus abdominis muscles [21] in patients with ALS is associated with their ventilatory dysfunction. However, no studies have investigated whether L1 levels of skeletal muscle can serve as a prognostic indicator for ALS. Therefore, we designed a cohort study hypothesizing that L1‐level skeletal muscles on chest CT reflect clinical severity and predict disease prognosis.

## 2 Methods

### 2.1 Participants

**FIGURE 1:**
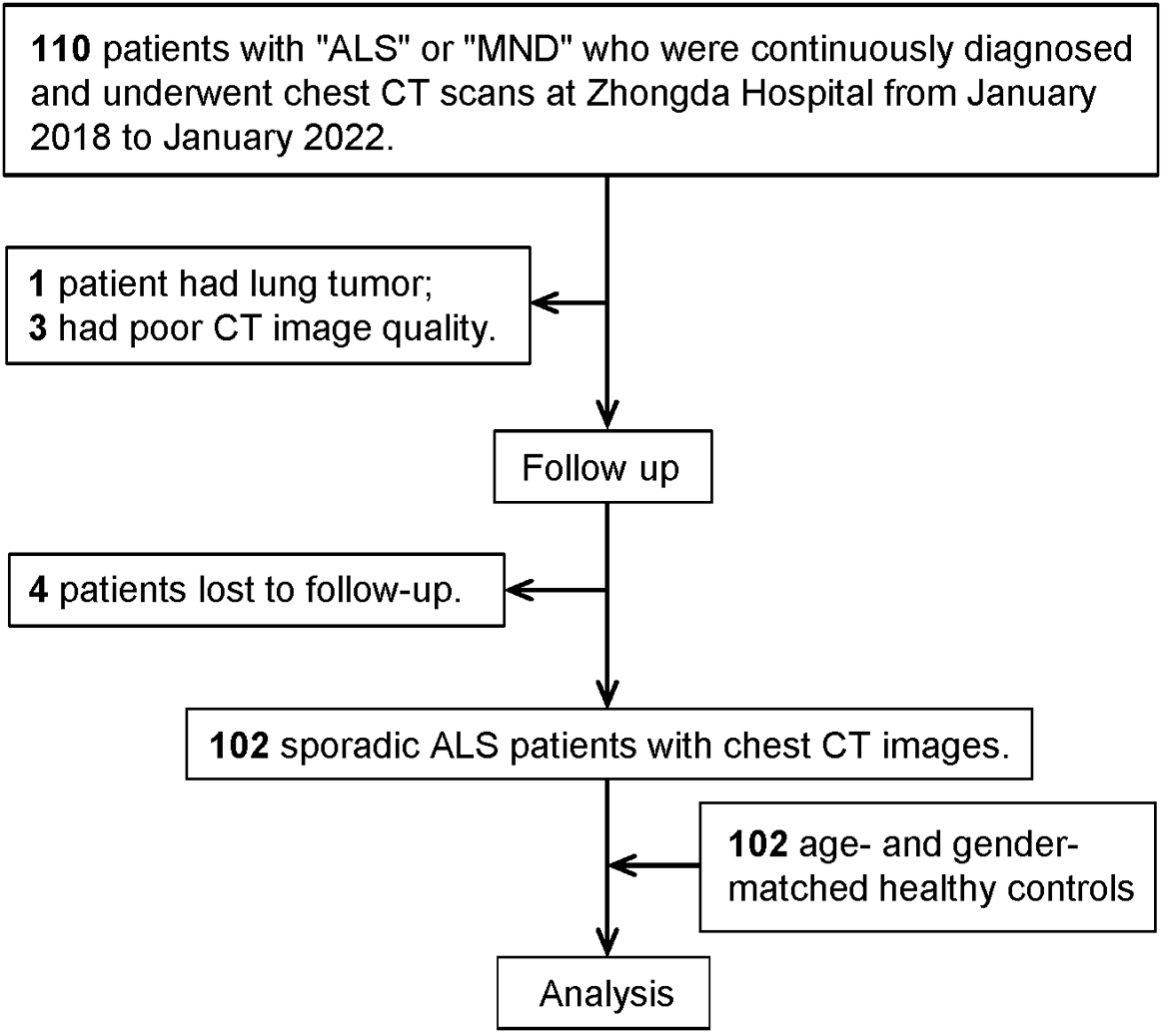
Study flowchart. A diagram of subject selection. ALS, amyotrophic lateral sclerosis; CT, computed tomography; MND, motor neuron disease.

We analysed the chest CT of consecutive patients diagnosed with ALS at Southeast University Affiliated Zhongda Hospital between January 2018 and January 2022. Diagnosis of definite, probable or possible ALS was based on the revised Awaji criteria. If multiple scans were available, we included the CT scan closest to ALS symptom onset. All enrolled patients were followed until death (inpatient or confirmed by phone follow‐up), tracheostomy or 1 January 2024. This defined overall survival (OS) after the chest CT. Finally, 102 ALS patients were included in the study after the exclusion of one case of concurrent tumour, three cases of poor CT image quality and four cases of patients lost to follow‐up (Figure 1). In total, 102 sex‐ and age‐matched healthy controls (HCs) were included from the physical examination centre. This study adheres to the STROBE reporting guidelines.

### 2.2 Demographic and Clinical Data

We collected the following demographic and clinical data from patient medical records, including sex, age, height, weight, BMI, date of onset, site of onset, Revised ALS Functional Rating Scale (ALSFRS‐R) score, ALSFRS‐R respiratory subgroup (ALSFRS‐R‐R), disease progression rate calculated as ([48 − ALSFRS‐R]/[time from onset to clinical evaluation]), King's clinical stages, smoking history and date of tracheostomy surgery. We collected data from ancillary examinations, including forced vital capacity (FVC), total protein (TP), albumin, creatine kinase (CK), serum creatinine (Scr), total cholesterol (TC), triglycerides (TG), high‐density lipoprotein (HDL) and low‐density lipoprotein (LDL) cholesterol, apolipoprotein A1 (apoA1), apolipoprotein B (apoB), lipoprotein a (Lpa) and haemoglobin A1C (HbA1C). In addition, we recorded the CT examination date and indications. Except for FVC, complete clinical data were available for 102 ALS patients. Only 40 patients had complete and valid FVC data.

The sample size of 102 patients adheres to the 10‐event‐per‐variable principle for Cox regression analysis, requiring 84–100 patients for the final calculation. Details regarding specific methods are provided in the Supporting Information.

### 2.3 CT Acquisition and Image Analysis

Chest CT examinations were performed using high‐resolution CT. Detailed scan parameters are provided in the Supporting Information.

To ensure interreader reproducibility and consistency in the slice section, two investigators (X. Jiang and C. Xie, with 3 and 9 years of imaging experience, respectively) reviewed chest CT scans from ALS patients and HCs using a standardized image viewing platform (Viewer, version 3.1; Neusoft PACS/RIS, Shenyang, China). They systematically identified, isolated and randomly assigned numbers to the CT slices that best represented the L1 vertebrae. Subsequently, the numbered slices were imported into ImageJ software (version 1.46; The National Institutes of Health, Bethesda, MD, USA) for semiautomated quantification of muscle and fat area by Y. Cao and D. Wu, following established methodologies from prior studies [22]. Different tissues were identified using Hounsfield unit (HU) thresholds: −29 to +150 for muscle and −190 to −30 for subcutaneous fat [23]. At the L1 vertebral level, SMA, SMD, SMI, PMA, paravertebral muscle density (PMD), subcutaneous fat area (SFA) and subcutaneous fat density (SFD) were calculated (Figure S1a–c). The detailed methodology for quantifying parameters is provided in the Supporting Information.

### 2.4 Genetic Testing

In total, 71 ALS patients underwent whole‐exome sequencing to identify pathogenic genes associated with ALS. We used a list of ALS‐related genes from the ALS online database and relevant published literature. Pathogenic mutations were defined according to the American College of Medical Genetics and Genomics recommendations. Detailed methodological descriptions are provided in the Supporting Information.

### 2.5 Statistics

Statistical methods included assessing normality with the Kolmogorov–Smirnov test, presenting continuous variables as mean ± standard deviation (SD) for normal distribution and median with interquartile range (IQR) for non‐normal distribution. Excluded were patients with missing data. Categorical variables were expressed as frequencies and percentages. Comparisons used *t*‐test for normally distributed continuous data, Mann–Whitney *U* test for non‐normal data and chi‐square test for categorical variables. ANOVA and post hoc tests analysed normal distributions across King's clinical stages; Kruskal–Wallis test and Bonferroni correction for non‐normal and ordered categorical variables. Spearman correlation assessed relationships. Restricted cubic spline (RCS) analysed non‐linear associations adjusting for covariates: sex; age; BMI for L1 SMA, L1 SMI and L1 PMA; and age for L1 PMD. Survival analysis defined endpoints as death or tracheostomy initiation. Cox regression identified predictors; *p* < 0.05 entered multivariate models. Training (*n* = 71) and validation (*n* = 31) cohorts tested the nomogram, assessing discrimination (concordance index [C‐index], area under the curve [AUC]), calibration (Hosmer–Lemeshow) and decision curve analysis (DCA). *p* < 0.05 indicates statistical significance; R software (v4.1.2) was used. Details of these methods are provided in the Supporting Information.

## 3 Results

### 3.1 Clinical Features

In total, 102 ALS patients (65 males; mean age, 60.85 ± 10.58 years) and age‐ and sex‐matched HCs were analysed (Table 1). There were 72 outcome events (41 tracheal intubations and 31 deaths). The median survival time following chest CT examination was 28.0 months (95% CI = 24.95–30.49). The mortality rates in the first and third years were 5.8% (95% CI = 0.01–0.10) and 21.4% (95% CI = 0.13–0.29), respectively. ALS patients demonstrated significantly lower L1 SMA, L1 PMA and L1 SMI, whereas they exhibited higher L1 SFA and L1 SFD compared with HCs (*p* < 0.001). Biochemical markers revealed lower TP, albumin and Scr levels but higher CK, apoA1 and Lpa levels in ALS patients compared with controls (*p* < 0.001; Table S1). Whole‐exome sequencing in 71 patients identified ALS‐associated risk genes in six patients (Table S2). Two women and one man experienced the outcome event, with respiratory failure caused by pulmonary infection identified as the cause of death. Women carrying ALS‐related gene mutations exhibited lower skeletal muscle mass. However, no significant differences were observed in quantitative skeletal muscle parameters between patients with and without ALS‐related gene mutations (Table S3).

**TABLE 1:** Demographic and clinical characteristics of the study participants.

Characteristic	ALS (*n* = 102)	HC (*n* = 102)	*t*/*Z*	*p*
Gender (M/F)	65/37	65/37		
Age at CT examination, year (mean ± SD)	60.85 ± 10.63	60.83 ± 10.36	*t* = −0.18	0.855
Diagnostic delay, month (median [IQR])	12 [6.25, 24]	—	—	—
ALSFRS‐R (mean ± SD)	38.09 ± 5.97	—	—	—
ALSFRS‐R‐R (median [IQR])	10 [9, 11.75]	—	—	—
King's clinical stage, *n* (%)		—	—	—
2	27 (26.47%)	—	—	—
3	44 (43.14%)	—	—	—
4a	9 (8.82%)	—	—	—
4b	22 (21.57%)	—	—	—
Smoking history, *n* (%)	35 (34.31%)	—	—	—
Site of onset, bulbar/nonbulbar	31/71			
BMI, kg/m^2^ (mean ± SD)	22.85 ± 3.06	23.59 ± 2.87	*t* = 1.78	0.076
L1 SMA, cm^2^ (mean ± SD)	85.25 ± 22.95	103.49 ± 17.71	*t* = 6.33	**< 0.001**
L1 SMD, HU (median [IQR])	35.91 [31.25, 41.2]	50 [46.25, 53]	*Z* = −8.32	**< 0.001**
L1 SMI, cm^2^/m^2^ (median [IQR])	30.3 [25.24, 35.59]	38.56 [35.28, 40.41]	*Z* = −7.38	**< 0.001**
L1 PMA, cm^2^ (mean ± SD)	35.41 ± 11.22	48.51 ± 8.86	*t* = 9.30	**< 0.001**
L1 PMD, HU (median [IQR])	38.56 [31.69, 45.24]	40.58 [37.1, 44.96]	*Z* = −2.02	**0.044**
L1 SFA, cm^2^ (median [IQR])	64.49 [44.94, 95.22]	54.11 [41.46, 65.32]	*Z* = −3.19	**0.001**
L1 SFD, HU (median [IQR])	−104.03 [−111.22, −94.72]	−110 [−113, −105]	*Z* = −4.01	**< 0.001**
FCV, *n* = 40 (mean ± SD)	86.58 ± 20.55	—	—	—

*Note: p*‐values inferior to 0.05 are reported in bold character.

Abbreviations: ALS, amyotrophic lateral sclerosis; ALSFRS‐R, ALS Functional Rating Scale‐Revised; ALSFRS‐R‐R, respiratory subgroup in ALSFRS‐R; BMI, body mass index; CT, computed tomography; F, female; FVC, forced vital capacity; HC, healthy controls; HU, Hounsfield units; IQR, interquartile range; L1, the first lumbar vertebra; L1 PMA, paravertebral muscle area at L1; L1 PMD, paravertebral muscle density at L1; L1 SFA, subcutaneous fat area at L1; L1 SFD, subcutaneous fat density at L1; L1 SMA, skeletal muscle area at L1; L1 SMD, skeletal muscle density at L1; L1 SMI, skeletal muscle index at L1; M, male; SD, standard deviation; *t*, *t*‐test; *Z*, Mann–Whitney test.

### 3.2 Association Between Clinical Features and Quantitative Parameters at L1 Level

**FIGURE 2:**
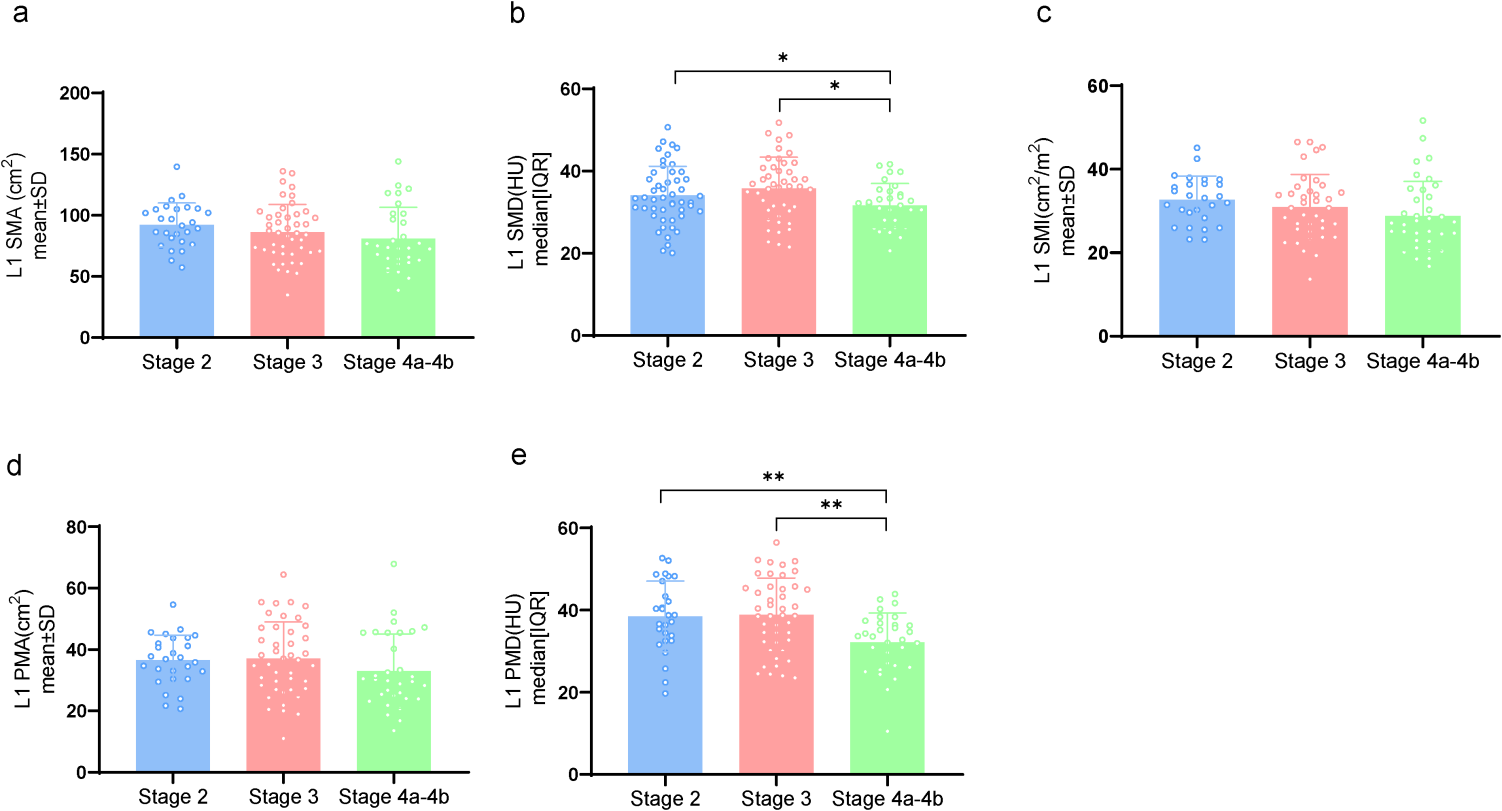
The first lumbar spine skeletal muscle and clinical stages. Associations between King's clinical stages and (a) L1 SMA, (b) L1 SMD, (c) L1 SMI, (d) L1 PMA and (e) L1 PMD. Normally distributed data (mean ± standard deviations) are analysed using one‐way analysis of variance and post hoc least significant difference tests. Non‐normally distributed data (median [interquartile range] are analysed using Kruskal–Wallis test and Bonferroni correction. *p < 0.05; **p < 0.01. CK, creatine kinase; HU, Hounsfield units; L1, first lumbar vertebra; L1 PMA, paravertebral muscle area at L1; L1 PMD, paravertebral muscle density at L1; L1 SMA, skeletal muscle area at L1; L1 SMD, skeletal muscle density at L1; L1 SMI, skeletal muscle index at L1; Scr, serum creatinine.

Quantitative analysis of skeletal muscle (Figure 2a–e) and subcutaneous fat (Figure S2a–b) at L1 revealed decreased values with increasing King's clinical stages. Notably, significant reductions were observed in L1 SMD and L1 PMD (Figure 2b,e, *p* < 0.05). We further identified significant sex‐based differences in L1 quantitative parameters. Compared with female ALS patients, male patients exhibited higher skeletal muscle mass, such as L1 SMA (*p* < 0.001), L1 SMI (*p* = 0.007) and L1 PMA (*p* < 0.009) (Table S4). However, their L1 SFA was significantly lower (*p* = 0.002). Compared with nonbulbar onset ALS, bulbar onset shows significantly lower skeletal muscle mass at L1 (SMD, *p* = 0.035) and higher fat mass (SFD, *p* = 0.016) (Table S4).

**FIGURE 3:**
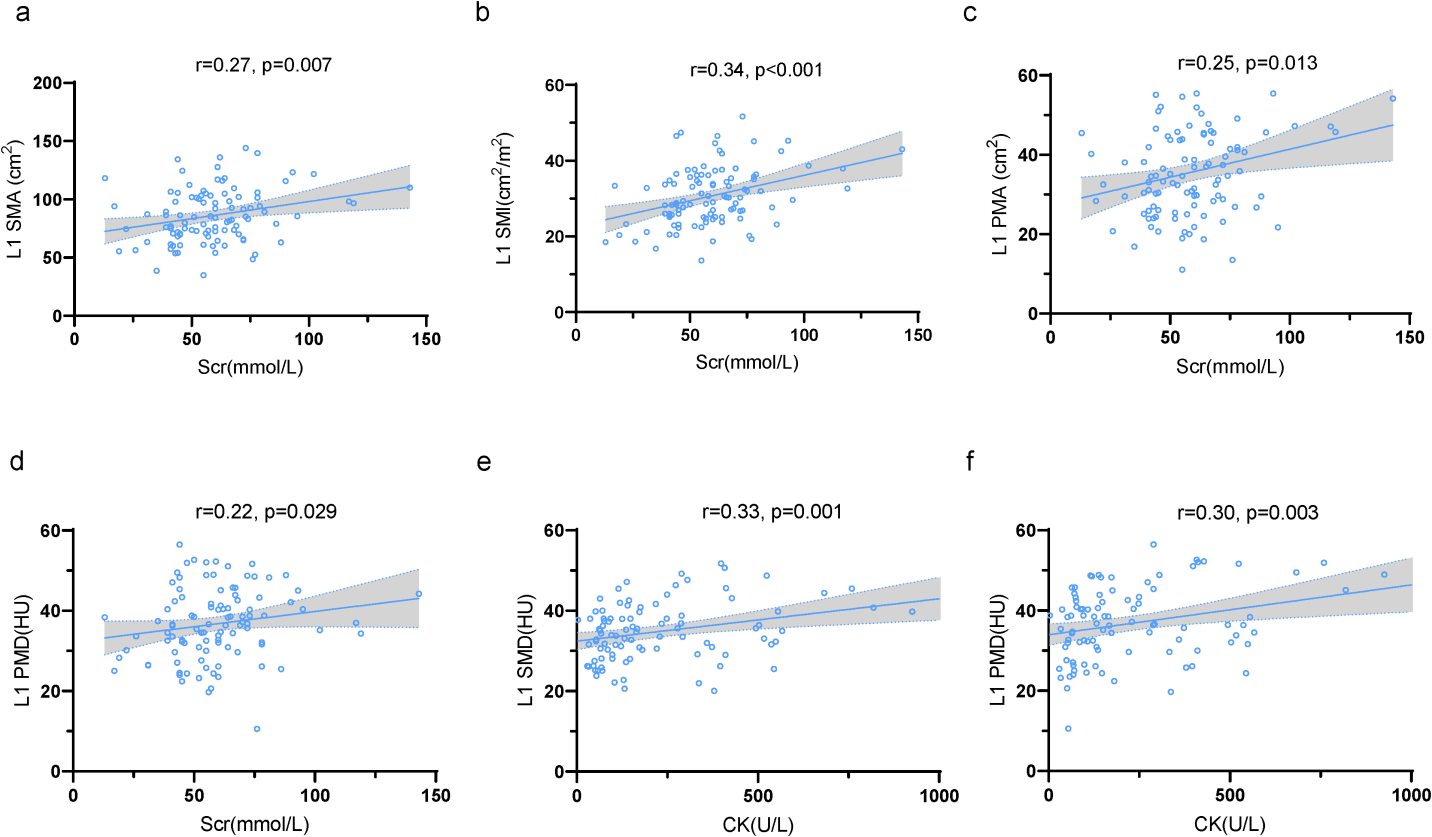
The first lumbar spine skeletal muscle and clinical severity. Associations between Scr and (a) L1 SMA, (b) L1 SMI, (c) L1 PMA, (d) L1 PMD; between CK and (e) L1 SMD, (f) L1 PMD; Spearman correlation. CK, creatine kinase; HU, Hounsfield units; L1, first lumbar vertebra; L1 PMA, paravertebral muscle area at L1; L1 PMD, paravertebral muscle density at L1; L1 SMA, skeletal muscle area at L1; L1 SMD, skeletal muscle density at L1; L1 SMI, skeletal muscle index at L1; Scr, serum creatinine.

Correlation analysis investigated the correlations between skeletal muscle parameters at the L1 level and clinical characteristics in ALS patients. Notably, L1 SMA (*r* = 0.27, *p* = 0.007), L1 SMI (*r* = 0.34, *p* < 0.001), L1 PMA (*r* = 0.25, *p* = 0.013) and L1 PMD (*r* = 0.22, *p* = 0.029) revealed positive correlations with Scr (Figure 3a–d). CK levels demonstrated positive correlations with L1 SMD (*r* = 0.33, *p* = 0.001) and L1 PMD (*r* = 0.30, *p* = 0.003) (Figure 3e–f), whereas ApoA1 levels were positively correlated with L1 SFA (Table S5). Similar positive correlations were observed with ALSFRS‐R, ALSFRS‐R‐R and BMI (Table S5). Age exhibited negative correlations with L1 SMD, L1 PMA and L1 PMD. No significant associations were observed between time to delayed diagnosis, time from symptom onset to CT, TP, albumin, TG, TC, HDL, LDL, ApoB, Lpa, HbA1C and skeletal muscle parameters (Table S5).

**FIGURE 4:**
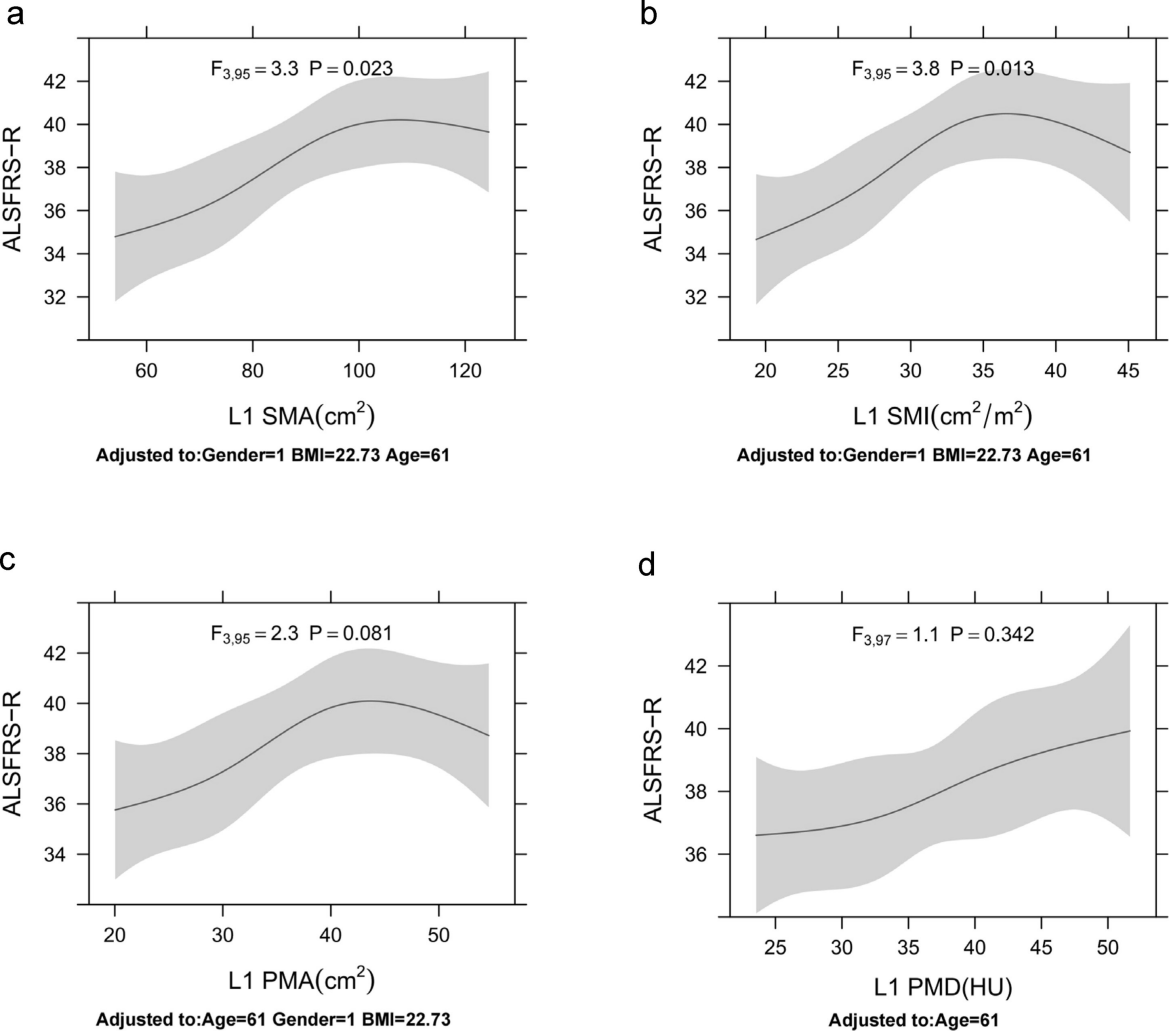
The first lumbar spine skeletal muscle and clinical severity. Associations between ALSFRS‐R and (a) L1 SMA, (b) L1 SMI, (c) L1 PMA and (d) L1 PMD. Restricted cubic spline with four knots was used for the independent variable. Analyses for L1 SMA, L1 SMI and L1 PMA were adjusted for age, sex and body mass index, whereas the analysis for L1 PMD was adjusted for age only. *p* < 0.05 indicates a statistically significant non‐linear relationship, whereas *p* > 0.05 indicates an approximately linear relationship. ALSFRS‐R, Amyotrophic Lateral Sclerosis Functional Rating Scale‐Revised; HU, Hounsfield units; L1, first lumbar vertebra; L1 PMA, paravertebral muscle area at L1; L1 PMD, paravertebral muscle density at L1; L1 SMA, skeletal muscle area at L1; L1 SMI, skeletal muscle index at L1.

To account for confounding factors, we visualized the associations between L1 SMA, L1 SMI, L1 PMA and L1 PMD and ALSFRS‐R and ALSFRS‐R‐R using RCS (Figures 3a–d and S3a–d). After adjusting for covariates, a non‐linear relationship was observed between ALSFRS‐R and L1 SMA (*F* = 3.3, *p* = 0.023) and L1 SMI (*F* = 3.8, *p* = 0.013). Notably, the strength of the association between L1 SMA and L1 SMI and ALSFRS‐R scores decreased when ALSFRS‐R scores < 40 (Figure 4a–b). ALSFRS‐R demonstrated a positive linear correlation with L1 PMA (*F* = 2.3, *p* = 0.081) and L1 PMD (*F* = 1.1, *p* = 0.342) (Figure 4c–d). Similarly, a non‐linear relationship was observed between ALSFRS‐R‐R and L1 SMI (*F* = 3.8, *p* = 0.012), with a decline in L1 SMI concordance when ALSFRS‐R‐R < 10 (Figure S3b). Conversely, ALSFRS‐R‐R exhibited a positive linear correlation with L1 SMA (*F* = 2.1, *p* = 0.101) and L1 PMA (*F* = 1.5, *p* = 0.225) (Figure S3a,c). Notably, L1 PMD (*F* = 1.4, *p* = 0.233) remained positively correlated with ALSFRS‐R‐R even after adjusting for age as a covariate (Figure S3d).

### 3.3 Survival Analysis and Nomogram Construction

**FIGURE 5:**
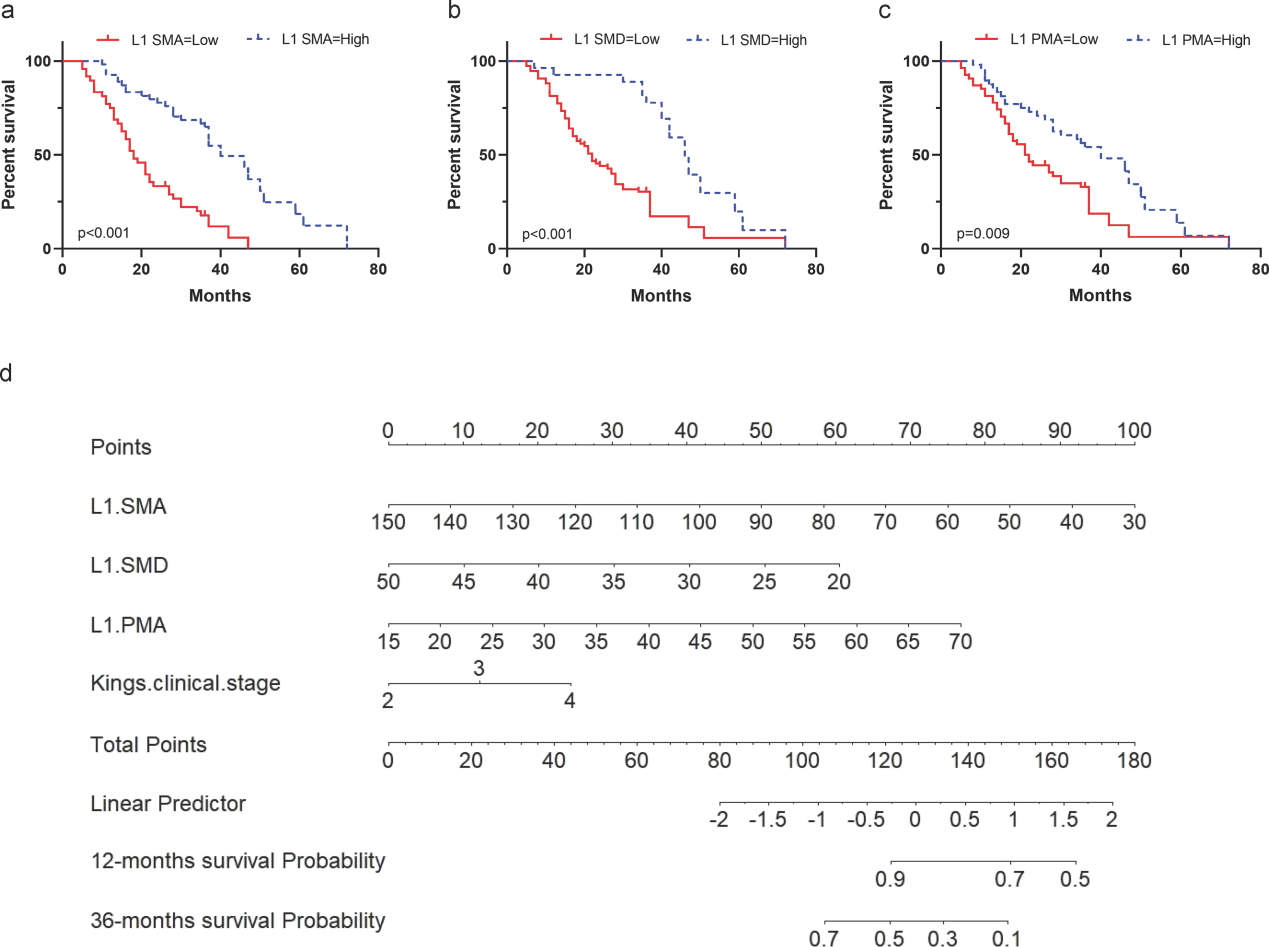
Survival analysis and nomogram of skeletal muscle parameters in ALS patients. Kaplan–Meier survival curve. (a) L1 SMA: Low < 81.53 cm^2^ and high ≥ 81.53 cm^2^; median survival = 18.0 vs. 40.0 months. (b) L1 SMD: Low < 38.75 HU and high ≥ 38.75 HU; median survival = 22.0 vs. 46.0 months. (c) L1 PMA: Low < 33.29 cm^2^ and high ≥ 33.29 cm^2^; median survival = 21.5 vs. 40.0 months. Stratified based on the threshold determined by the maximum selection test. Log‐rank test. (d) Nomogram construction using the ALS training dataset and independent predictors. HU, Hounsfield units; L1, first lumbar vertebra; L1 PMA, paravertebral muscle area at L1; L1 SMA, skeletal muscle area at L1; L1 SMD, skeletal muscle density at L1.

Univariate Cox regression analysis identified the following statistically significant indicators for survival (*p* < 0.05): ALSFRS‐R, King's clinical stages, BMI, site of onset, L1 SMA, L1 SMD, L1 SMI, L1 PMA and L1 PMD (Table 2). In the subsequent multivariate analysis, King's clinical stages (Stage 3 HR = 2.3, 95% CI = 1.15–4.59, *p* = 0.018; Stage 4a–b HR = 3.43, 95% CI = 1.64–7.16, *p* = 0.001), L1 SMA (HR = 0.96, 95% CI = 0.94–0.98, *p* = 0.001), L1 SMD (HR = 0.92, 95% CI = 0.88–0.95, *p* < 0.001) and L1 PMA (HR = 1.06, 95% CI = 1.01–1.11, *p* = 0.022) were identified as independent prognostic factors affecting survival (Table 2). Considering that gender may affect disease progression and survival in ALS, we included it in the multivariate Cox regression analysis (Table S6). Consistent with previous findings, gender did not influence the risk of mortality in our cohort. After verifying that the Cox model satisfied the necessary assumptions using the residual method (Figure S4a–c), we identified the optimal thresholds for survival‐related L1 SMA (81.53 cm^2^), L1 SMD (38.75 HU) and L1 PMA (33.29 cm^2^) (Figure S5a–c). Kaplan–Meier curves demonstrated statistically significant differences in OS between groups stratified by L1 SMA, L1 SMD and L1 PMA thresholds (Figure 5a–c, *p* < 0.01).

**TABLE 2:** Univariable and multivariable Cox regression analysis in ALS patients.

	Univariable		Multivariable model	
	HR [95% CI]	*p*	HR [95% CI]	*p*
Age	1.01 [0.99, 1.03]	0.203	—	—
Gender (female)	1.36 [0.83, 2.24]	0.228	—	—
Course of disease	1.00 [0.99, 1.01]	0.808	—	—
ALSFRS‐R	0.93 [0.90, 0.97]	**< 0.001**	—	—
King's clinical stages				
2	—	—	—	—
3	2.12 [1.07, 4.20]	**0.031**	2.30 [1.15, 4.59]	**0.018**
4a–4b	5.23 [2.61, 10.51]	**< 0.001**	3.43 [1.64, 7.16]	**0.001**
Site of onset (bulbar)	2.28 [1.36, 3.80]	**0.002**	—	—
L1 SMA, cm^2^	0.98 [0.97, 0.99]	**< 0.001**	0.96 [0.94, 0.98]	**0.001**
L1 SMD, HU	0.92 [0.89, 0.95]	**< 0.001**	0.92 [0.88, 0.95]	**< 0.001**
L1 SMI, cm^2^/m^2^	0.94 [0.91, 0.97]	**< 0.001**	—	—
L1 PMA, cm^2^	0.97 [0.95, 0.99]	**0.008**	1.06 [1.01, 1.11]	**0.022**
L1 PMD, HU	0.94 [0.91, 0.96]	**< 0.001**	—	—
L1 SFA, cm^2^	1.00 [0.99, 1.00]	0.506	—	—
L1 SFD, HU	1.00 [0.98, 1.02]	0.702	—	—
BMI, kg/m^2^	0.94 [0.87, 1.01]	0.093	—	—
Diagnostic delay	0.99 [0.96, 1.02]	0.554	—	—

*Note:* Survival time was defined as the time from the chest CT scan to the functional endpoint (death or tracheostomy). *p*‐values inferior to 0.05 are reported in bold character.

Abbreviations: ALSFRS‐R, Amyotrophic Lateral Sclerosis Functional Rating Scale‐Revised; BMI, body mass index; CI, confidence interval; HR, hazard ratio; HU, Hounsfield units; L1, the first lumbar vertebra; L1 PMA, paravertebral muscle area at L1; L1 PMD, paravertebral muscle density at L1; L1 SFA, subcutaneous fat area at L1; L1 SFD, subcutaneous fat density at L1; L1 SMA, skeletal muscle area at L1; L1 SMD, skeletal muscle density at L1; L1 SMI, skeletal muscle index at L1.

**FIGURE 6:**
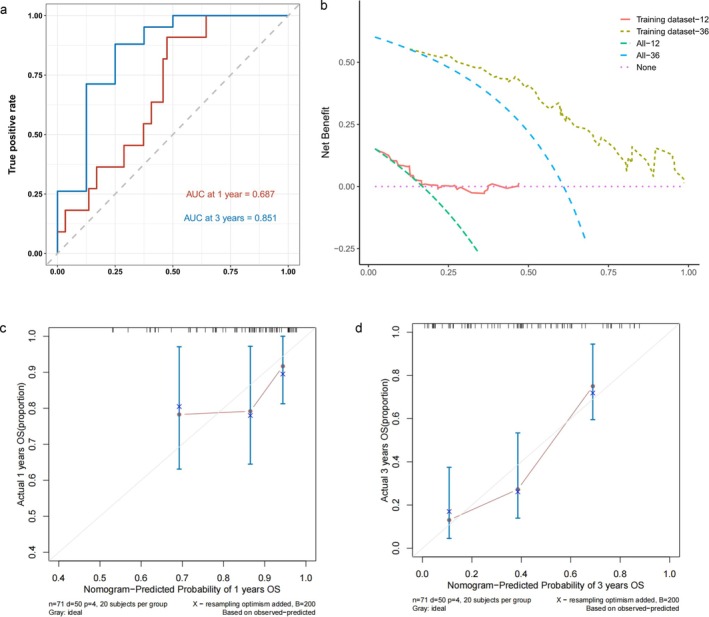
Evaluation of nomogram model. (a) Receiver operating characteristic curves for predicting 1‐ and 3‐year survival. (b) Decision curve analysis curves for predicting 12‐ and 36‐month survival calibration curves for predicting 1‐year (c) and 3‐year (d) survival rates of the training set cohort.

A nomogram was developed, integrating all independent predictors of OS from the training set (Figure 5d). The C‐index for the nomogram was 0.72 (95% CI = 0.641–0.793). The nomogram assigns points to each parameter based on a point scale. The sum of these points provides a total score, which can be used to estimate OS probabilities. This practical approach allows for a more intuitive the 1‐ and 3‐year OS prediction in ALS patients. The AUCs predicted by the nomogram model for 1‐ and 3‐year OS are 0.687 and 0.851, respectively (Figure 6a). Furthermore, DCA curves indicated the superior efficacy of the model in predicting 3‐year OS (Figure 6b). Calibration curves in the training set further supported the agreement between predicted and actual OS outcomes (Figure 6c–d), suggesting moderate performance of the nomogram in predicting ALS OS sensitivity and specificity. Internal validation yielded a C‐index of 0.85 (95% CI = 0.777–0.932) for OS in the validation set. The AUCs for 1‐ and 3‐year OS prediction were 0.907 and 0.992, respectively (Figure S6a). Calibration and DCA curves in the validations set corroborated these findings (Figure S6b–d). Consequently, independent predictors, including King's clinical stages, L1 SMA, L1 SMD and L1 PMA, hold promise for predicting ALS prognosis.

## 4 Discussion

Our study investigated the use of chest CT to measure muscle and fat at the L1 level in ALS patients. The findings demonstrate that skeletal muscle quantity at L1 can predict outcomes in ALS. We confirmed the presence of reduced muscle mass in ALS patients and established that total skeletal muscle and paraspinal muscle at L1 can predict survival. In addition, we identified a correlation of muscle mass with the functional status and disease stage in patients with ALS, whereas subcutaneous fat changed solely in relation to the disease stage.

In this exploratory study, we identified associations between overall and local muscle area/density and survival outcomes in ALS patients, suggesting a potential prognostic role for L1‐level skeletal muscle parameters. However, further validation in larger, ALS‐specific cohorts is required to confirm these findings and establish their clinical utility. Clinically, muscle atrophy in affected areas often receives primary focus, neglecting the evaluation of overall skeletal muscle status. Recent studies suggest a positive contribution of peripheral tissues, particularly skeletal muscles, to ALS pathogenesis [5]. These findings support the hypothesis that ALS comprehensively affects overall skeletal muscles. The reduction in SMD (mean attenuation density) observed on CT is typically associated with an increase in intramuscular adipose tissue [24]. ALS patients have lower SMD, indicating the myosteatosis, which is associated with their poorer clinical functional status and poorer prognosis. Therefore, myosteatosis has relevant implications in both the pathology and clinical management of ALS. Skeletal muscle MRI in ALS suggests that changes in local muscle MRI‐related parameters can serve as potential biomarkers for ALS symptom severity [25]. Diamanti et al. demonstrated more severe paravertebral muscle steatosis using MRI in ALS patients, with a bulbar‐onset predominance, consistent with our findings [26]. We investigated a larger cohort of ALS patients to further elucidate the relationship between overall and paravertebral skeletal muscle characteristics, clinical features and prognosis. Bauckneht et al., using positron emission tomography‐CT, observed significantly lower psoas muscle volume in ALS patients with high mortality [19]. Our findings suggest that a lower area of L1 paraspinal muscles (including the psoas major muscle) is associated with higher mortality rates, yielding consistent results. In addition, Choi et al., using deep learning analysis of abdominal CT, revealed a decrease in SMI with advancing disease stage [27]. However, no significant associations were observed between quantitative skeletal muscle parameters and ALS survival prognosis. This discrepancy might be attributed to variations in skeletal muscle function at different lumbar levels. Pickhardt et al., in a large CT deep learning study, reported that muscle area and density at the L1 level were more effective predictors of survival compared with other lumbar vertebrae, potentially reflecting functional heterogeneity within the lumbar paravertebral muscles [28]. Our study preliminarily explored the relationship between skeletal muscle parameters at L1 and L3 levels in ALS patients (Figure S7). Although the small sample size (*n* = 15) limits definitive conclusions, we observed a strong correlation between L1 and L3 muscle data, consistent with findings of Derstine BA et al. in a larger cohort [16]. Incorporating L3, a common site for sarcopenia assessment, into future studies could enhance predictive models of ALS progression. The addition of multiple muscle levels would offer a more comprehensive understanding of muscle atrophy and its role in disease progression, potentially leading to more accurate ALS prognostic tools. Subcutaneous fat at the L1 level is partially correlated with the disease stage; nonetheless, it is not related to the progression and prognosis of ALS‐related diseases. Therefore, comprehensive management strategies, including proper nutrition, appropriate rehabilitation and the avoidance of muscle‐toxic drugs, can provide long‐term benefits for patients. Despite its limitations, the nomogram offers a valuable clinical tool. Its intuitive visual format enables physicians and patients to better understand and predict disease progression. Notably, it emphasizes the need to integrate assessments of overall and paravertebral muscle status in ALS patients.

Our study identified SMD as a key independent predictor in the prognostic model of ALS. Myosteatosis intramuscular fat accumulation is the final common pathological pathway for many different primary genetic and acquired neuromuscular disorders [29]. Previous studies have suggested that ALS patients have significantly higher accumulation of muscle fibre lipid droplets, which are associated with lipid metabolism disorders and mitochondrial dysfunction [30]. As the respiratory function score of ALS patients decreases and King's clinical stages progress, there is a significant decrease in L1‐level skeletal muscle indicators. This is consistent with the varying degrees of respiratory dysfunction that gradually appear in ALS patients in the middle and late stages. The decline in muscle density in this study may further support the pathological pattern of muscle mass degradation and infiltration by adipose/fibrous tissue, suggesting that muscle imaging parameters may serve as dynamic monitoring indicators of disease progression. Furthermore, the low muscle density and high fat mass characteristics of patients with bulbar onset are associated with earlier respiratory dysfunction. Impaired swallowing function may result in inadequate early nutrient intake, thereby accelerating muscle loss; meanwhile, this increases the risk of infection, as inflammation may promote myosteatosis [31]. Consistent moderate exercise paired with tailored nutritional interventions, particularly incorporating dietary antioxidants such as vitamin D, vitamin A and retinoic acid, may effectively reduce muscle fat accumulation while simultaneously increasing muscle mass and strength [31]. These interventions warrant systematic evaluation in ALS cohorts to determine their impact on myosteatosis and functional outcomes. Quantitative imaging biomarkers such as L1‐level SMD may provide objective measures of myosteatosis, which could inform clinical assessments of disease progression.

The reduction of muscle mass may be affected by various factors, including gender, age, malnutrition and physical inactivity, all of which affect the progression and survival of ALS. These findings to some extent reflect the complexity of muscle degeneration in ALS, and genetic factors alone may not fully explain the variability of disease progression. Our results show that women with ALS‐related gene mutations tend to have lower skeletal muscle mass. Integrating genetic information with advanced imaging techniques could offer a more comprehensive understanding of how ALS‐related gene mutations affect muscle degeneration and progression. Such an approach would allow for the identification of subtle phenotypic changes that may not be captured by standard skeletal muscle quantification methods, providing more granular insights into disease mechanisms. On the other hand, the influence of heavy metals on the muscle tissue warrants careful attention. Among them, cadmium (Cd) exposure is associated with an increased risk of all‐cause mortality and ALS [32, 33]. Chronic Cd exposure induces skeletal muscle injury by upregulating pro‐apoptotic genes, disrupting lipid metabolism and enhancing inflammation through pro‐inflammatory cytokine production [34], all of which may accelerate the progression of muscular atrophy in ALS. Notably, Cd is not only an external toxicant but also released from skeletal muscle during atrophy, further exacerbating its detrimental effects [35]. This bidirectional relationship between Cd toxicity and muscle degeneration underscores the need for further research to elucidate its precise role in ALS‐related muscle deterioration.

In addition, CT‐based quantitative methods effectively distinguish between skeletal muscle and adipose tissue. This distinction is supported by the correlation between skeletal muscle parameters and Scr and CK levels. CK, a serum enzyme elevated in approximately 40% of ALS patients, reflects muscle denervation and altered fibre metabolism [36]. Conversely, decreased Scr levels in ALS likely correlate with reduced muscle mass and diminished Scr uptake, mirroring disease progression [37]. In addition, several studies demonstrate a decline in Scr associated with fat‐free mass reduction in ALS patients, suggesting its utility for monitoring changes in fat‐free mass [38]. Therefore, our rapid and straightforward quantitative method offers robust differentiation between muscle and fat.

Our study has several limitations. First, CT radiation exposure necessitates careful considerations, as it limits the frequency of skeletal muscle assessments. Second, the single‐centre study design and relatively small sample size restrict the generalizability of our findings and hinder more in‐depth analysis. Our findings may not provide valuable clinical insights for patients with ALS‐related genetic mutations, thus warranting a multicentre prospective study with a larger cohort. Therefore, a multicentre, prospective study with a larger cohort is warranted. Third, the prognostic value of single‐level skeletal muscle quantification is limited. Integrating multilevel assessments and specific muscle group segmentation may enhance ALS progression and survival prediction models. Fourth, our cross‐sectional design precludes causal conclusions between L1 muscle parameters and ALS prognosis, as observed associations may reflect confounding factors (e.g., disease severity and genetics) rather than causation. Longitudinal studies with serial imaging are needed to determine if L1 muscle decline drives or mirrors disease progression. Future work should integrate multimodal data (genetic, inflammatory and respiratory metrics) to clarify L1 muscle–ALS interactions.

In conclusion, our study demonstrates a significant association between progressive reductions in overall skeletal muscle mass and L1‐level paravertebral skeletal muscle mass with ALS disease progression and poorer survival outcomes. These findings emphasize the value of skeletal muscle quantitative assessments in chest CT for prognostic evaluation in ALS. They offer insights into new prognosis monitoring indicators for patients.

## Ethics Statement

The present study was carried out in accordance with the latest version of the Declaration of Helsinki. The study was approved by the Ethics Committee of Zhongda Hospital, School of Medicine, Southeast University (2019ZDKYSB098, 2022ZDSYLL289‐P01). All ALS patients and healthy subjects who underwent the study signed an informed consent form. All authors of this manuscript certify that they comply with the ethical guidelines for authorship and publishing in the *Journal of Cachexia, Sarcopenia and Muscle* [39].

## Conflicts of Interest

The authors declare no conflicts of interest.

## Supporting information


**Table S1** Laboratory examinations of research participants.
**Table S2** Clinical features of patients carrying scattered ALS gene mutations.
**Table S3** Clinical characteristics of genetic and non‐genetic ALS patients.
**Table S4** The relationship between gender, site of onset and CT quantitative parameters.
**Table S5** Association between clinical features and quantitative parameters at L1 level.
**Table S6** Univariable and multivariable Cox regression analysis in ALS patients.
**Figure S1** Region of interest at the first lumbar vertebra within the chest computed tomography. (a) Skeletal muscle area; (b) paravertebral muscle area; (c) subcutaneous fat area.
**Figure S2** The first lumbar spine (L1) subcutaneous fat and clinical stages. Relationship between King’s clinical stages and (a) L1 SFA; (b) L1 SFD; mean ± SD, one‐way ANOVA and LSD tests; median [IQR], Kruskal–Wallis tests and Bonferroni corrections. *p < 0.05. L1 SFA, subcutaneous fat area at L1; L1 SFD, subcutaneous fat density at L1; HU, Hounsfield units.
**Figure S3** The first lumbar spine skeletal muscle and respiratory function severity. Associations between ALSFRS‐R‐R and (a) L1 SMA, (b) L1 SMI, (c) L1 PMA and (d) L1 PMD. Restricted cubic spline with four knots was used for the independent variable. Analyses for L1 SMA, L1 SMI and L1 PMA were adjusted for age, sex and body mass index, whereas the analysis for L1 PMD was adjusted for age only. *p*‐values < 0.05 indicate a statistically significant non‐linear relationship, whereas *p*‐values > 0.05 indicate an approximately linear relationship. ALSFRS‐R‐R, Amyotrophic Lateral Sclerosis Functional Rating Scale‐Revised respiratory subgroup; L1, first lumbar vertebra; L1 SMA, skeletal muscle area at L1; L1 SMI, skeletal muscle index at L1; L1 PMA, paravertebral muscle area at L1; L1 PMD, paravertebral muscle density at L1; HU, Hounsfield units.
**Figure S4** Cox proportional risk regression models for L1 SMA, L1 PMD and King’s clinical stages. (a) Standardized (scaled) Schoenfeld residuals are independent of survival time, indicating the proportional risk assumption is satisfied; (b) deviance residuals are dispersed and symmetric; (c) Martingale residual plots and partial residual plots are approximately linear.
**Figure S5** The maximum‐choice log‐rank test was used to determine thresholds for quantitative CT metrics, identifying the optimal thresholds for survival: (a) L1 SMA was 81.53 cm^2^; (b) L1 SMD was 38.75 cm^2^; and (c) L1 PMA was 33.29 HU. L1, first lumbar vertebra; L1 SMA, skeletal muscle area at L1; L1 PMA, paravertebral muscle area at L1; L1 PMD, paravertebral muscle density at L1; HU, Hounsfield units.
**Figure S6** Validation set cohort: (a) receiver operating characteristic (ROC) curves of 1 and 3 years; (b) decision curve analysis (DCA) curves of 12 and 36 months; calibration curves of 1 year (c) and 3 years (d). OS, overall survival.
**Figure S7** Association between skeletal muscle parameters at L1 level and L3 level. L1, the first lumbar vertebra; L3 = the third lumbar vertebra; SMA = skeletal muscle area; SMD, skeletal muscle density; SMI = skeletal muscle index; PMA, paravertebral muscle area; PMD, paravertebral muscle density; Spearman correlation.

## Data Availability

The data sets generated or analysed during the study are available from the corresponding authors on reasonable request.
