# Supplementary material for: Quantification of Skeletal Muscle at the First Lumbar Level for Prognosis in Amyotrophic Lateral Sclerosis
Source: J Cachexia Sarcopenia Muscle. 2025 Jun 5;16(3):e13827. doi: 10.1002/jcsm.13827 (PMC12138266; doi:10.1002/jcsm.13827)
Supplement: Supplementary file 1 — Table S1 Laboratory examinations of research participants. Table S2 Clinical features of patients carrying scattered ALS gene mutations. Table S3 Clinical characteristics of genetic and non‐genetic ALS patients. Table S4 The relationship between gender, site of onset and CT quantitative parameters. Table S5 Association between clinical features and quantitative parameters at L1 level. Table S6 Univariable and multivariable Cox regression analysis in ALS patients. Figure S1 Region of interest at the first lumbar vertebra within the chest computed tomography. (a) Skeletal muscle area; (b) paravertebral muscle area; (c) subcutaneous fat area. Figure S2 The first lumbar spine (L1) subcutaneous fat and clinical stages. Relationship between King’s clinical stages and (a) L1 SFA; (b) L1 SFD; mean ± SD, one‐way ANOVA and LSD tests; median [IQR], Kruskal–Wallis tests and Bonferroni corrections. *p < 0.05. L1 SFA, subcutaneous fat area at L1; L1 SFD, subcutaneous fat density at L1; HU, Hounsfield units. Figure S3 The first lumbar spine skeletal muscle and respiratory function severity. Associations between ALSFRS‐R‐R and (a) L1 SMA, (b) L1 SMI, (c) L1 PMA and (d) L1 PMD. Restricted cubic spline with four knots was used for the independent variable. Analyses for L1 SMA, L1 SMI and L1 PMA were adjusted for age, sex and body mass index, whereas the analysis for L1 PMD was adjusted for age only. p‐values < 0.05 indicate a statistically significant non‐linear relationship, whereas p‐values > 0.05 indicate an approximately linear relationship. ALSFRS‐R‐R, Amyotrophic Lateral Sclerosis Functional Rating Scale‐Revised respiratory subgroup; L1, first lumbar vertebra; L1 SMA, skeletal muscle area at L1; L1 SMI, skeletal muscle index at L1; L1 PMA, paravertebral muscle area at L1; L1 PMD, paravertebral muscle density at L1; HU, Hounsfield units. Figure S4 Cox proportional risk regression models for L1 SMA, L1 PMD and King’s clinical stages. (a) Standardized (scaled) Schoenfeld residuals [file JCSM-16-e13827-s001.docx]

**Online** **supplementary material**

**Quantification of Skeletal Muscle at the First Lumbar Level for Prognosis in** **Amyotrophic Lateral Sclerosis**

Yujia Cao, MD^1,2^; Baoyu Yuan, PhD^1,2^, Xiuyu Jiang, MD^2^, Chunming Xie, PhD ^1,2^, Di Wu, PhD ^1,2*^, Zhijun Zhang, PhD ^1,2*^

1.Department of Neurology, Affiliated ZhongDa Hospital, School of Medicine, Institution of Neuropsychiatry, Key Laboratory of Developmental Genes and Human Disease, Southeast University, Nanjing, Jiangsu 210009, China.

2.Jiangsu Key Laboratory of Molecular and Functional Imaging, Department of Radiology, Zhongda Hospital, School of Medince ,Southeast University, Nanjing, China.

* Address correspondence to Zhijun Zhang, Ph.D., E-mail: janemengzhang@vip.163.com. Di Wu, Ph.D., E-mail: doctor.[diwu@gmail.com](mailto:diwu@gmail.com).

**Supplemental Methods**

**Sample Size Calculation and Indications for Chest CT**

To determine the appropriate sample size for a Cox proportional hazards regression analysis, it is essential to ensure that the study is sufficiently powered to detect a significant effect.^1^ For ALS, a significant factor is the three-year mortality rate, which typically ranges from 50% to 60% based on scientific literature.^2^ Let's use an average event rate of 55% for our calculation. A common guideline is a minimum of 10 events per covariate. The total sample size required to achieve the required number of events was estimated to be 84 to 100, based on the mortality event rate in the ALS population.

Pre-cancer screening was the primary indication for chest CT, accounting for 50.98% (52/102) of patients. Among them, small pulmonary nodules were detected in 43.14% (44/102) of patients, while ground-glass opacities were found in 4.90% (5/102). No significant abnormalities were observed in 2.94% (3/102) of cases. Lung infections constituted the second most common reason for chest CT, with pneumonia diagnosed in 38.24% (39/102) of patients, and bronchiectasis with superimposed infection in 7.84% (8/102). Chest tightness and trauma were less frequent indications, accounting for 1.96% (2/102) and 0.98% (1/102) of cases, respectively.

**Genetic Testing**

Peripheral venous blood samples were collected from the ALS patients, and DNA was isolated from mononuclear cells using a DNA isolation kit (Blood DNA Kit V2, CW2553). Principles of ALS gene selection the genes of *SOD1, ALS2, SETX, FUS, VAPB, TARDBP, OPTN, VCP, UBQLN2, SIGMAR1, FIG4, CHMP2B, PFN1, NEFH, PRPH, TFG, TAF15, GRN, CHCHD10, TUBA4A, TBK1, NEK1, GLE1, MATR3, CCNF, ANXA11, HNRNPA1, SQSTM1, ERBB4, TIA1, SPG11,* and *KIF5A* were screened for causative mutations. Besides those commonly accepted ALS genes, only frameshift or nonsense mutations in the following genes were defined as pathogenic: *NEK1, GLE1, NEFH, CHCHD10*, and *TUBA4A*. The frequency of mutations was determined using gnome AD and exoneme aggregation consortium to exclude common single nucleotide polymorphisms. Only non-synonymous, splicing, and frameshift variants with minor allele frequencies (MAF) below 0.5% from all population databases were selected for further analysis. The procedure comprised three standard steps: end-­repair of fragmented DNA, A-­tailing, and adapter ligation and amplification. Sequencing was completed by the HiSeq2000 platform (Illumina, San Diego, California, USA) using 90 bp paired-­end reads. The sequence was aligned to the human reference genome (UCSC hg19) using a Burrows-­Wheeler Aligner and then reformatted using SAM tools. ^3^ Variant frequencies were initially determined in gnomeAD and Exome Aggregation Consortium to remove the common single nucleotide polymorphisms. Only non-­synonymous, splicing and frameshift variants with a MAF of less than 0.5% across all population databases were selected for further analysis. Variant annotation and filtering were performed by Mutation Taster (http://www. mutationtaster. org), ^4^ SIFT (http:// sift. jcvi. org/), ^5^ and PolyPhen2 (http:// genetics. bwh. harvard. edu/ pph2/).^6^ Mutations identified by WES were further validated by Sanger sequencing. *C9orf72* repeat amplification was screened using standard repeat primer PCR. ^7^And predicting criteria for high-penetrance mutations centered on most acknowledged areas including genetic, pathology, functional studies and informatics analyses.

**CT Acquisition Protocol and Image Analysis**

CT examinations were performed using high-resolution CT (Discovery CT750 HD Scanner, GE Healthcare, GE Medical System, Zhongda Hospital, Southeast University, ct99; Helical Scanning Mode; software version sles_hde3.5). Chest CT scan parameters were defined as follows: tube voltage of 120 kVp, tube current of 260 mA under automatic exposure control, and a rotation time of 1.0 seconds. The CT scan was reconstructed with a section width of 5 mm.

The identification of different tissues was achieved through the utilization of Hounsfield unit (HU) boundaries, which were set from -29 to +150 for muscle and -190 to -30 for subcutaneous fat. ^8^The methodology employed in previous studies is referenced.^9^ The specific procedures were as follows: A) L1 SMA: The ImageJ software was used to adjust the threshold range between -29 and 150 HU to cover all muscles within the region of interest (ROI) and automatically correct the boundaries. The boundaries of the ROIs were then manually corrected by selecting muscle groups, including the psoas major, erector spinae, quadratus lumborum, latissimus dorsi, transversus abdominis, internal obliques, and lateral abdominal muscles.^10^ The size of the generated ROIs was measured in square centimeters (Figure. S1a). Simultaneously, the average density within the ROI, known as the mean attenuation density (L1 SMD in HU), was calculated and recorded. The L1 SMI was obtained by dividing the L1 SMA by the square of the subject's height (cm²/m²). B) L1 PMA: The bilateral paravertebral muscles were distinguished by manually correcting the borders of the ROI using ImageJ, based on L1 SMA. The PMA primarily includes the erector spinae and quadratus lumborum.^10^ Subsequently, the ROI area was automatically generated (Figure. S1b), and the mean density within the ROI was recorded as L1 PMD. C) L1 SFA: The boundaries of the ROI were manually corrected to distinguish between subcutaneous and visceral fat, and the ROI area was generated by adjusting the HU range between -190 and -30 on the same image plane (Figure. S1c). The mean density within the ROI is the L1 SFD.

A total of 15 patients underwent abdominal CT scans during the medical visit. We systematically identify, separate, and number the CT slices of the same patient that best represent the L1 and L3 vertebrae. The same method was used to quantify the skeletal muscle area (L3 SMA), skeletal muscle density (L3 SMD), and skeletal muscle index (L3 SMI) at the L3 level.

**Statistics**

Patients with missing data were excluded from statistical analyses. Restricted cubic spline (RCS) function was used to analyze the nonlinear relationship between ALSFRS-R, ALSFRS-R-R, and CT quantitative parameters, adjusting for relevant covariates. Sex, age, and BMI were included as covariates for L1 SMA, L1 SMI, and L1 PMA, whereas only age was adjusted for L1 PMD. In the survival analysis, the functional endpoint was defined as death from any cause or initiation of tracheostomy dependent ventilation. The maximum-choice log-rank test was used to establish optimal cut-off points for continuous independent predictors. Subsequently, Kaplan-Meier curves were generated based on these thresholds. Log-rank tests were used to compare survival differences between groups in the Kaplan-Meier analysis. To ensure the development of the model, patients were randomly assigned to training (n=71) and internal validation (n=31) queues using the "sample" function, with a ratio of 7:3. The accuracy of the nomogram was evaluated using the discriminate ability of the training and validation sets, along with a calibration curve. In addition, the consistency index (C-index) and area under the receiver operating characteristic curve (AUC) were used to evaluate the final predictive model. A calibration curve was constructed and validated using the Hosmer-Lemeshow test to assess model calibration. In addition, decision curve analysis (DCA) was used to evaluate the clinical utility of the nomogram by calculating net benefits across various threshold probabilities. A 2-tailed p-values < 0.05 were considered statistically significant. Statistical analyses were performed using R software (version 4.1.2).

**Supplementary References**

1. Chow SC, Shao J, Wang H. Sample Size Calculations in Clinical Research. 2nd Edition. Chapman & Hall/CRC.; 2008
2. Hardiman O, Al-Chalabi A, Chio A, Corr EM, Logroscino G, Robberecht W et al. Amyotrophic lateral sclerosis. Nat Rev Dis Prim 2017; 3:17071.
3. Etherington GJ, Ramirez-Gonzalez RH, MacLean D. bio-samtools 2: a package for analysis and visualization of sequence and alignment data with SAMtools in Ruby. Bioinformatics (Oxford, England). 2015;31(15):2565-2567.
4. Schwarz JM, Cooper DN, Schuelke M, Seelow D. MutationTaster2: mutation prediction for the deep-sequencing age. Nat Methods. 2014;11(4):361-362.
5. Kumar P, Henikoff S, Ng PC. Predicting the effects of coding non-synonymous variants on protein function using the SIFT algorithm. Nat Protoc. 2009;4(7):1073-1081.
6. Adzhubei IA, Schmidt S, Peshkin L, Ramensky VE, Gerasimova A, Bork P, Kondrashov AS, Sunyaev SR. A method and server for predicting damaging missense mutations. Nat Methods. 2010;7(4):248-249.
7. Richards S, Aziz N, Bale S, Bick D, Das S, Gastier-Foster J et al. Standards and guidelines for the interpretation of sequence variants: a joint consensus recommendation of the American College of Medical Genetics and Genomics and the Association for Molecular Pathology. Genet Med Off J Am Coll Med Genet 2015; 17:405–424.
8. Irving BA, Weltman JY, Brock DW, Davis CK, Gaesser GA, Weltman A. NIH ImageJ and Slice-O-Matic computed tomography imaging software to quantify soft tissue. Obesity 2007; 15:370–376.
9. Teigen LM, Kuchnia AJ, Nagel E, Deuth C, Vock DM, Mulasi U et al. Impact of Software Selection and ImageJ Tutorial Corrigendum on Skeletal Muscle Measures at the Third Lumbar Vertebra on Computed Tomography Scans in Clinical Populations. J Parenter Enter Nutr 2018; 42:933–941.
10. Dixon AK, Bowden DJ, Logan BM, Ellis H. Human Sectional Anatomy: Pocket atlas of body sections, CT and MRI images. 4th ed. CRC Press; 2017:141-142.

**Supplemental Tables**

| **Table S1. Laboratory Examinations of Research Participants.** | | | | | |
| --- | --- | --- | --- | --- | --- |
| **Characteristic** | | **ALS(n=102)** | **HC(n=102)** | **t/Z** | ***p*** |
| TP, g/L (mean ± SD) | 64.35 ± 6.28 | 72.35 ± 4.16 | t= 10.70 | **<0.001** |  |
| Albumin, g/L (median [IQR]) | 39.8 [38,43.6] | 43[40, 46] | Z= -4.34 | **<0.001** |  |
| CK, IU/L, n=95(median [IQR]) | 147 [75,335] | 44.20[40.6, 49.4] | Z= -10.05 | **<0.001** |  |
| Scr, μmol/L (median [IQR]) | 57.5 [45,68.75] | 70.72[61.88, 79.56] | Z= -5.57 | **<0.001** |  |
| TG, mmol/L (median [IQR]) | 1.29 [0.88,1.86] | 1.34 ± 1.28 | t= -1.47 | 0.143 |  |
| TC, mmol/L (mean ± SD) | 4.5 ± 1.23 | 4.91 [4.06, 5.38] | Z= -1.58 | 0.115 |  |
| HDL, mmol/L (median [IQR]) | 1.26 [1.02,1.46] | 1.27 [1.14, 1.58] | Z= -1.71 | 0.087 |  |
| LDL, mmol/L (mean ± SD) | 2.66 ± 0.89 | 2.69 ± 0.84 | t= 0.21 | 0.834 |  |
| ApoA1, g/L (median [IQR]) | 1.17 [1.01,1.42] | 0.99 [0.77, 1.67] | Z= -2.09 | 0.036 |  |
| ApoB, g/L (median [IQR]) | 0.86 [0.67,1.02] | 0.84 [0.68, 1.04] | Z= -0.49 | 0.621 |  |
| Lpa, mg/L (median [IQR]) | 156 [76.5,285.5] | 70.72[61.88, 88.4] | Z= -5.76 | **<0.001** |  |
| HbA1c (median [IQR]) | 5.4 [5.1,6.07] | 5.40 [5.2, 5.6] | Z= -0.86 | 0.392 |  |
| Abbreviations: TP, Total Protein; CK, creatine ki-se; Scr, serum creatinine; TC, total cholesterol; TG, triglycerides; H/LDL, high/low-density lipoprotein cholesterol; ApoA1, Apolipoprotei-1; ApoB, Apolipoprotein B; Lpa, Lipoprotein a; HbA1c, hemoglobin A1c; t, t-test; Z, Mann-Whitney test. p-values inferior to 0.05 are reported in bold character. | | | | |  |

| **Table S2 Clinical features of patients carrying scattered ALS gene mutations.** | | | | | | | | | | | |
| --- | --- | --- | --- | --- | --- | --- | --- | --- | --- | --- | --- |
| **Sex** | **Age, year** | **Clinical phenotype** | **Mutant gene, Nucleotide change** | **Amino acid change** | **L1 SMA, cm^2^** | **L1 SMD, HU** | **L1 PMA, cm^2^** | **L1 PMD, HU** | **L1 SMI, cm²/m²** | **Overall survival, month** | **End point** |
| M | 35 | LMN | *SOD1*, c.32G>C | p. F21C | 127.83 | 42.04 | 51.94 | 41.26 | 37.35 | 48 | - |
| F | 52 | LMN | *C9orf72* | - | 75.15 | 28.03 | 33.29 | 24.38 | 29.36 | 19 | death |
| F | 58 | LMN | *ANG*, c.223C>T | p. R75C | 84.77 | 31.64 | 32.89 | 35.41 | 33.11 | 36 | - |
| M | 60 | UMN | *ERBB4*, c.2407T>G | p.C803G | 110.10 | 40.03 | 54.19 | 44.23 | 43.01 | 36 | death |
| F | 57 | Bulbar paralysis | *NEFH*, c.856A>C | p. Thr286Pro | 69.71 | 32.87 | 50.99 | 32.74 | 26.98 | 8 | death |
| F | 50 | LMN | *VCP*, c.410C>T | p. P137L | 63.06 | 47.16 | 29.53 | 48.90 | 23.16 | 47 | - |
| Abbreviations: M=male; F=female; LMN=lower motor neuron; UMN=upper motor neuron; L1= The First Lumbar Vertebra; L1 SMA= Skeletal Muscle Area at L1; L1 SMD= Skeletal Muscle Density at L1; L1 SMI= Skeletal Muscle Index at L1; L1 PMA= Paravertebral Muscle Area at L1; L1 PMD= Paravertebral Muscle Density at L1. | | | | | | | | | | | |

| **Table S3 Clinical characteristics of genetic and non-genetic ALS patients.** | | | | |
| --- | --- | --- | --- | --- |
| **Variables** | **Non-genetic ALS (n = 65)** | **Genetic ALS (n = 6)** | **Statistic** | ***p*** |
|  |  |  |  |  |
| Age, Mean ± SD | 61.06 ± 10.28 | 66.50 ± 5.05 | t=-1.27 | 0.207 |
| Men/women (n) | 41/24 | 4/2 | χ²=0.00 | 1 |
| Site Of Onset, n (%) |  |  | χ²=0.00 | 1 |
| bulbar | 45 (69.23) | 4 (66.67) |  |  |
| Non-bulbar | 20 (30.77) | 2 (33.33) |  |  |
| BMI, Mean ± SD | 23.04 ± 2.91 | 25.47 ± 3.07 | t=-1.95 | 0.056 |
| Time from diagnosis to CT scan (median [IQR]) | 12.00 (7.00, 24.00) | 11.00 (10.00, 12.00) | Z=-0.28 | 0.78 |
| ALSFRS-R (median [IQR]) | 39.00 (35.00, 41.00) | 39.00 (35.00, 42.25) | Z=-0.13 | 0.893 |
| King’s Clinical Stage, n (%) |  |  | - | 0.796 |
| 2 | 17 (26.15) | 2 (33.33) |  |  |
| 3 | 25 (38.46) | 2 (33.33) |  |  |
| 4a | 5 (7.69) | 1 (16.67) |  |  |
| 4b | 18 (27.69) | 1 (16.67) |  |  |
| L1 SMA, cm2(mean ± SD) | 84.23 ± 19.81 | 86.35 ± 24.74 | t=-0.25 | 0.807 |
| L1 SMD, HU (median [IQR]) | 34.97[29.16, 39.81] | 31.26[31.01, 36.34] | Z=-0.80 | 0.426 |
| L1 SMI, cm²/m2(mean ± SD) | 29.07[25.53, 33.85] | 31.67[27.17, 35.93] | Z=-0.64 | 0.522 |
| L1 PMA, cm2(mean ± SD) | 34.99 ± 10.41 | 38.95 ± 11.17 | t=-0.89 | 0.378 |
| L1 PMD, HU (mean ± SD) | 36.61[30.92, 43.93] | 35.10[33.03, 40.20] | Z=-0.31 | 0.756 |
| L1 SFA, cm2(median [IQR]) | 59.62 [42.14, 97.28] | 66.78 [60.21, 76.69] | Z=-0.28 | 0.78 |
| L1 SFD, HU (median [IQR]) | -104.00 [-112.72, -92.78] | -103.03 [-107.22, -101.48] | Z=-0.28 | 0.78 |
| Abbreviations: ALS, amyotrophic lateral sclerosis; SD, standard deviation; IQR, interquartile range; CT , computed tomography; ALSFRS-R , ALS Functional Rating Scale-Revised; BMI , body mass index; L1, The First Lumbar Vertebra; L1 SMA, Skeletal Muscle Area at L1; L1 SMD, Skeletal Muscle Density at L1; L1 SMI, Skeletal Muscle Index at L1; L1 PMA, Paravertebral Muscle Area at L1; L1 PMD, Paravertebral Muscle Density at L1; L1 SFA, Subcutaneous Fat Area at L1; L1 SFD, Subcutaneous Fat Density at L1; HU, Hounsfield Units; t, t-test; Z, Mann-Whitney test. | | | | |

| **Table S4 The relationship between gender, site of onset, and CT quantitative parameters.** | | | | | | | |
| --- | --- | --- | --- | --- | --- | --- | --- |
| **Variable** | **Gender** | | | **Site of onset** | | | |
|  | **Male**  **(n=65)** | **Female**  **(n=37)** | **p** |  | **Non-bulbar**  **(n=71)** | **Bulbar**  **(n=31)** | ***p*** |
| L1SMA, cm^2^(mean ±SD) | 92.39±22.14 | 75.04±19.94 | **<0.001** | 88.74±22.53 | | 80.03±22.80 | 0.077 |
| L1SMD, HU (median [IQR]) | 35.45[31.16,39.81] | 32.17[28.03,38] | 0.081 | 35.62[30.52,40.41] | | 32.78[27.12,35.84] | **0.035** |
| L1 SMI, cm²/m² (median [IQR]) | 32.04[26.88,37.35] | 26.98[24.74,32.83] | **0.007** | 31.49[25.74,36.16] | | 27.39[25.10,33.55] | 0.221 |
| L1PMA, cm^2^ (mean ±SD) | 37.79±11.60 | 31.81±9.12 | **0.009** | 36.41±10.93 | | 33.79±11.40 | 0.279 |
| L1PMD, HU (median [IQR]) | 37.16[32.13,43.93] | 34.73[27.64,40.39] | 0.21 | 36.65[32.01,43.81] | | 35.17[27.65,40.28] | 0.173 |
| L1SFA, cm^2^(median [IQR]) | 57.09[41.69, 82.96] | 93.58[56.65,120.23] | **0.002** | 68.18[49.04,95.10] | | 52.27[28.2,97.56] | 0.141 |
| L1SFD, HU (median [IQR]) | 104.00[ -113.32, -95.49] | -104.41[-109.58, -94.50] | 0.958 | -105.17[-113.57, -97.17] | | -97.90[-113.57, -87.26] | **0.016** |
| Abbreviations: SD=standard deviation; IQR=inter-quartile range, L1= The First Lumbar Vertebra; L1 SMA= Skeletal Muscle Area at L1; L1 SMD= Skeletal Muscle Density at L1; L1 SMI= Skeletal Muscle Index at L1; L1 PMA= Paravertebral Muscle Area at L1; L1 PMD= Paravertebral Muscle Density at L1; L1 SFA= Subcutaneous Fat Area at L1; L1 SFD= Subcutaneous Fat Density at L1; HU= Hounsfield Units. t-test, Mann-Whitney test. p-values inferior to 0.05 are reported in bold character. | | | | | | | |

| Table S5 Association between clinical features and quantitative parameters at L1 level. | | | | | | | |
| --- | --- | --- | --- | --- | --- | --- | --- |
|  | L1SMA, cm^2^ | L1SMD, HU | L1 SMI, cm**²**/m**²** | L1PMA, cm^2^ | L1PMD, HU | L1SFA, cm^2^ | L1SFD, HU |
| Age at CT examination, year | -0.13 (p=0.18) | -0.31 (**p=0.001**) | -0.14 (p=0.172) | -0.21 (**p=0.035**) | -0.24 (**p=0.015**) | -0.00 (p=0.97) | -0.04 (p=0.688) |
| Diagnostic delay, month | -0.19 (p=0.061) | -0.15 (p=0.129) | -0.16 (p=0.107) | -0.19 (p=0.051) | -0.16 (p=0.121) | 0.08 (p=0.438) | -0.14 (p=0.171) |
| ALSFRS-R | 0.35 (**p=0.000**) | 0.19 (p=0.054) | 0.34 (**p=0.000**) | 0.27 (**p=0.007**) | 0.20 (**p=0.048**) | 0.17 (p=0.083) | 0.042 (p=0.672) |
| ALSFRS-R-R | 0.27 (**p=0.006**) | 0.19 (p=0.055) | 0.32 (**p=0.001**) | 0.22 (**p=0.028**) | 0.24 (**p=0.015**) | 0.22 (**p=0.026**) | -0.03 (p=0.768) |
| BMI | 0.24 (**p=0.016**) | 0.10 (p=0.304) | 0.26 (**p=0.008**) | 0.14 (p=0.151) | 0.03 (p=0.736) | 0.33 (**p=0.001**) | -0.13 (p=0.185) |
| Time from diagnosis to CT scan, month | -0.01 (p=0.912) | -0.05 (p=0.604) | -0.02 (p=0.826) | -0.13 (p=0.2) | -0.06 (p=0.544) | -0.01 (p=0.962) | 0.17 (p=0.082) |
| Total Protein, g/L | 0.01 (p=0.926) | 0.01 (p=0.94) | 0.07(p=0.465) | 0.03 (p=0.76) | -0.04 (p=0.731) | 0.21 (p=0.035) | -0.04 (p=0.727) |
| Albumin, g/L | 0.13 (p=0.192) | 0.03 (p=0.733) | 0.19 (p=0.052) | 0.13 (p=0.194) | 0 (p=0.998) | -0.06 (p=0.535) | 0.13 (p=0.192) |
| CK, IU/L, n=95 | 0.13 (p=0.213) | 0.33 (**p=0.001**) | 0.13 (p=0.197) | 0.12 (p=0.262) | 0.30 (**p=0.003**) | -0.11 (p=0.309) | 0.06 (p=0.598) |
| Creatinine, umol/L | 0.27 (**p=0.007**) | 0.18 (p=0.064) | 0.34 (**p=0.000**) | 0.25 (**p=0.013**) | 0.22 (**p=0.029**) | -0.06 (p=0.525) | 0.07 (p=0.508) |
| TG, mmol/L | -0.11 (p=0.271) | 0.01 (p=0.889) | -0.04 (p=0.663) | -0.01 (p=0.894) | 0.01 (p=0.952) | 0.08 (p=0.456) | -0.10 (p=0.315) |
| TC, mmol/L | -0.01 (p=0.933) | 0.02 (p=0.814) | 0.04 (p=0.714) | 0.01 (p=0.959) | -0.04 (p=0.721) | 0.10 (p=0.317) | -0.03 (p=0.791) |
| HDL, mmol/L | -0.11 (p=0.266) | -0.04 (p=0.708) | -0.09 (p=0.39) | -0.13 (p=0.209) | -0.05 (p=0.624) | 0.10 (p=0.326) | 0.01 (p=0.934) |
| LDL, mmol/L | 0.02 (p=0.837) | 0.04 (p=0.732) | 0.07 (p=0.502) | 0.01 (p=0.927) | -0.01 (p=0.926) | 0.10 (p=0.33) | 0.01 (p=0.892) |
| Apolipoprotein A, g/L | -0.09 (p=0.392) | -0.12 (p=0.233) | -0.05 (p=0.628) | -0.09 (p=0.358) | -0.14 (p=0.169) | 0.28 (**p=0.005**) | -0.15 (p=0.141) |
| Apolipoprotein B, g/L | -0.07 (p=0.525) | -0.00 (p=0.993) | -0.01 (p=0.887) | -0.02 (p=0.816) | -0.04 (p=0.697) | 0.11 (p=0.292) | -0.14 (p=0.157) |
| Lipoprotein a, mg/L | 0.16 (p=0.118) | -0.07 (p=0.522) | 0.13 (p=0.19) | 0.14 (p=0.18) | -0.11 (p=0.271) | 0.16 (p=0.11) | -0.19 (p=0.057) |
| HbA1c | 0.09 (p=0.398) | 0.15 (p=0.131) | 0.18 (p=0.068) | 0.10 (p=0.328) | 0.17 (p=0.085) | 0.06 (p=0.525) | 0.12 (p=0.233) |

Abbreviations: ALSFRS-R = Amyotrophic Lateral Sclerosis Functional Rating Scale-Revised; ALSFRS-R-R=respiratory subgroup in ALSFRS-R; BMI = body mass index; FVC = forced vital capacity; TP=Total Protein; CK=creatine kinase; TC= total cholesterol; TG=triglycerides; H/LDL= high/low-density lipoprotein cholesterol; HbA1c = hemoglobin A1c. L1= The First Lumbar Vertebra; L1 SMA= Skeletal Muscle Area at L1; L1 SMD= Skeletal Muscle Density at L1; L1 SMI= Skeletal Muscle Index at L1; L1 PMA= Paravertebral Muscle Area at L1; L1 PMD= Paravertebral Muscle Density at L1; L1 SFA= Subcutaneous Fat Area at L1; L1 SFD= Subcutaneous Fat Density at L1; HU= Hounsfield Units. p-values inferior to 0.05 are reported in bold character. Spearman correlation test.

**Table S6. Univariable and Multivariable Cox Regression Analysis in ALS patients.**

|  | | **Uni-variable** | | | |  | | **Multi-variable model 2** | | | |
| --- | --- | --- | --- | --- | --- | --- | --- | --- | --- | --- | --- |
|  | | **HR [95%CI]** | | **P** | |  | | **HR [95%CI]** | | **P** | |
| Age | | 1.01[0.99,1.03] | | 0.203 | |  | | - | | - | |
| Gender(female) | | 1.36[0.83,2.24] | | 0.228 | |  | | - | | - | |
| Course of disease | | 1.00[0.99,1.01] | | 0.808 | |  | | - | | - | |
| ALSFRS-R | | 0.93[0.90,0.97] | | <0.001 | |  | | - | | - | |
| King's clinical stages | |  | |  | |  | |  | |  | |
| 2 | | - | | - | |  | | - | | - | |
| 3 | | 2.12[1.07,4.20] | | 0.031 | |  | | 2.45[1.25,4.81] | | 0.009 | |
| 4a-4b | | 5.23[2.61,10.51] | | <0.001 | |  | | 2.96[1.41,6.21] | | 0.004 | |
| Site of onset (Bulbar) | | 2.28[1.36,3.80] | | 0.002 | |  | | - | | - | |
| L1 SMA, cm^2^ | | 0.98[0.97,0.99] | | <0.001 | |  | | 0.96[0.94,0.98] | | 0.001 | |
| L1 SMD, HU | | 0.92[0.89,0.95] | | <0.001 | |  | | 0.91[0.88,0.95] | | <0.001 | |
| L1 SMI, cm²/m² | | 0.94[0.91,0.97] | | <0.001 | |  | | - | | - | |
| L1 PMA, cm^2^ | | 0.97[0.95,0.99] | | 0.008 | |  | | 1.06[1.01,1.11] | | 0.023 | |
| L1 PMD, HU | | 0.94[0.91,0.96] | | <0.001 | |  | | - | | - | |
| L1 SFA, cm^2^ | | 1.00[0.99,1.00] | | 0.506 | |  | | - | | - | |
| L1 SFD, HU | | 1.00[0.98,1.02] | | 0.702 | |  | | - | | - | |
| BMI, kg/m^2^ | | 0.94[0.87,1.01] | | 0.093 | |  | | - | | - | |
| Diagnostic delay | | 0.99[0.96,1.02] | | 0.554 | |  | | - | | - | |

Survival time was defined as the time from the chest CT scan to the functional endpoint (death or tracheostomy). Inclusion of single factor regression with p<0.05 and gender factor in multiple factor regression. Abbreviations: HR, hazard ratio; CI, confidence interval; ALSFRS-R , Amyotrophic Lateral Sclerosis Functional Rating Scale-Revised; BMI , body mass index; L1, The First Lumbar Vertebra; L1 SMA, Skeletal Muscle Area at L1; L1 SMD, Skeletal Muscle Density at L1; L1 SMI, Skeletal Muscle Index at L1; L1 PMA, Paravertebral Muscle Area at L1; L1 PMD, Paravertebral Muscle Density at L1; L1 SFA, Subcutaneous Fat Area at L1; L1 SFD, Subcutaneous Fat Density at L1; HU, Hounsfield Units.

**Supplemental Figures**

| 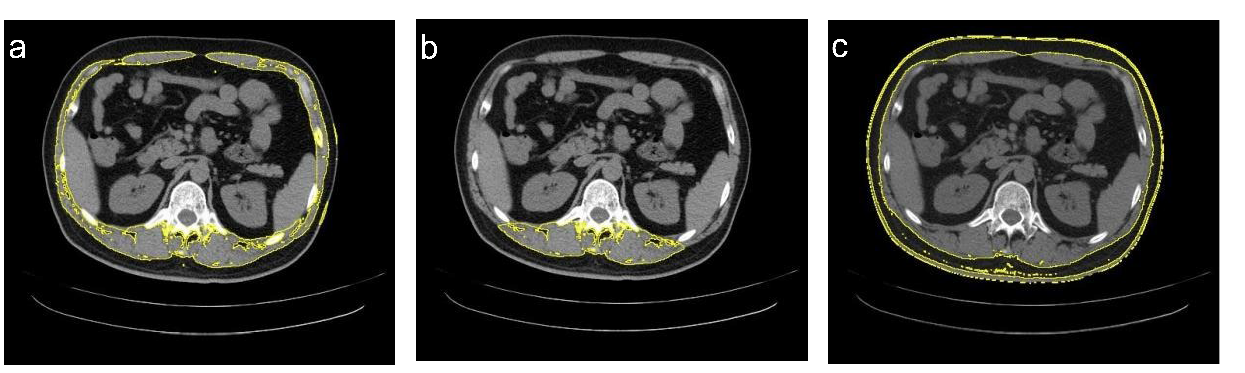 |  |  |
| --- | --- | --- |
| **Figure S1. Region of Interest at the First Lumbar Vertebra within the chest computed tomography.** (a) Skeletal muscle area; (b) paravertebral muscle area; (c) subcutaneous fat area. | | |


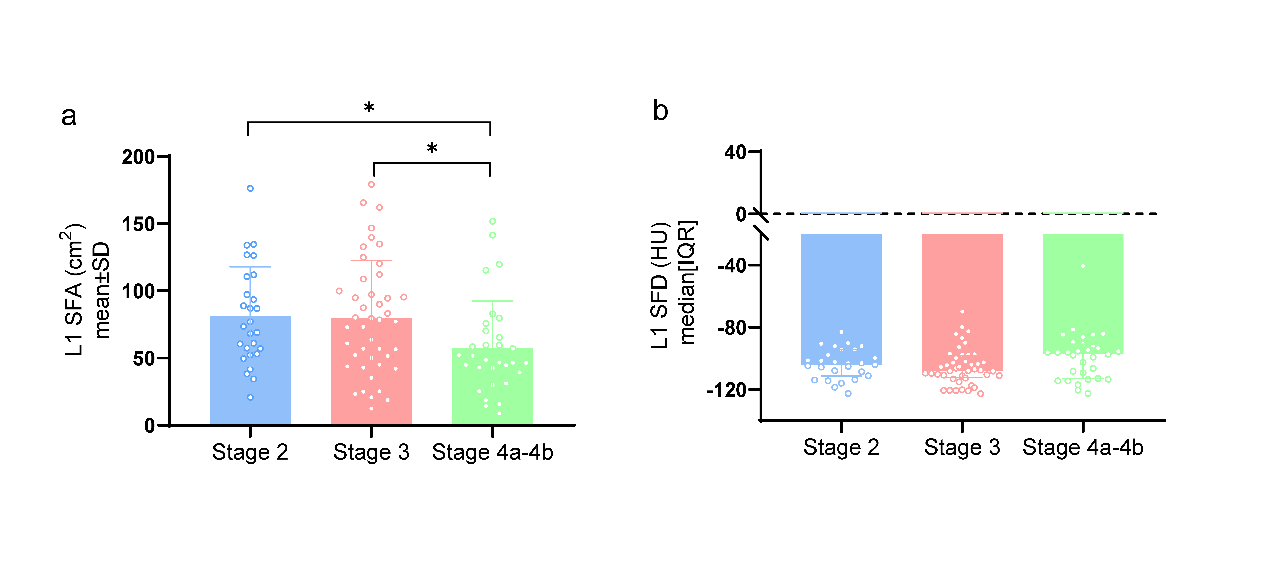


**Figure S2. The First Lumbar Spine(L1) subcutaneous fat and Clinical Stages.** Relationship between King's clinical stages and (a) L1 SFA; (b) L1 SFD; Mean ± SD, one-way ANOVA, and LSD tests; median [IQR], Kruskal-Wallis tests, and Bonferroni corrections. *P < 0.05. L1 SFA, subcutaneous fat area at L1; L1 SFD, subcutaneous fat density at L1, HU, Hounsfield Units.


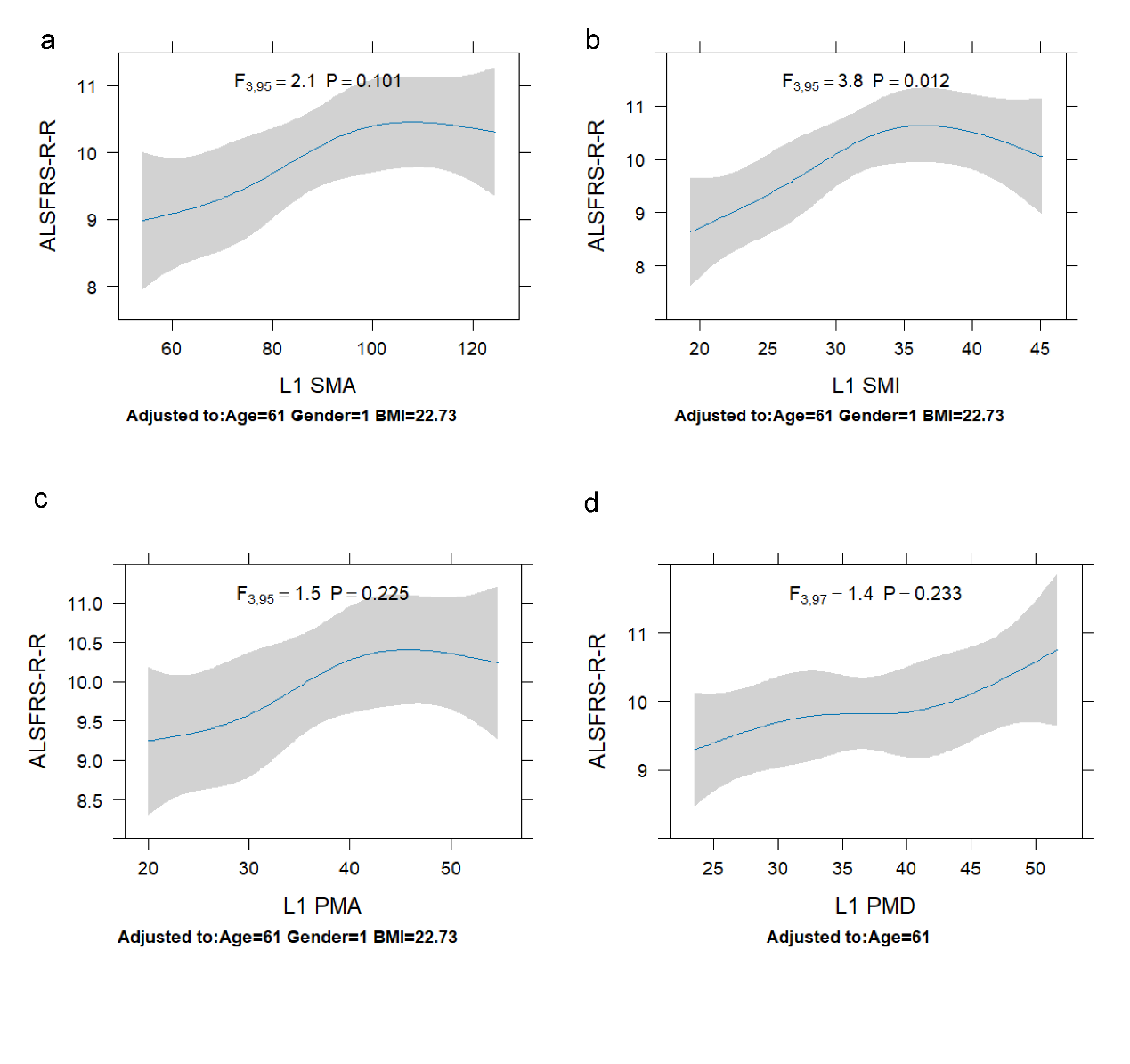


**Figure S3. The First Lumbar Spine Skeletal Muscle and Respiratory Function Severity.** Associations between ALSFRS-R-R and (a) L1 SMA, (b) L1 SMI, (c) L1 PMA, and (d) L1 PMD. Restricted cubic spline with 4 knots was used for the independent variable. Analyses for L1 SMA, L1 SMI, and L1 PMA were adjusted for age, sex, and body mass index, whereas the analysis for L1 PMD was adjusted for age only. P values < 0.05 indicate a statistically significant nonlinear relationship, whereas P values > 0.05 indicate an approximately linear relationship. ALSFRS-R-R, Amyotrophic Lateral Sclerosis Functional Rating Scale-Revised respiratory subgroup; L1, first lumbar vertebra; L1 SMA, skeletal muscle area at L1; L1 SMI, skeletal muscle index at L1; L1 PMA, paravertebral muscle area at L1; L1 PMD, paravertebral muscle density at L1; HU, Hounsfield Units.


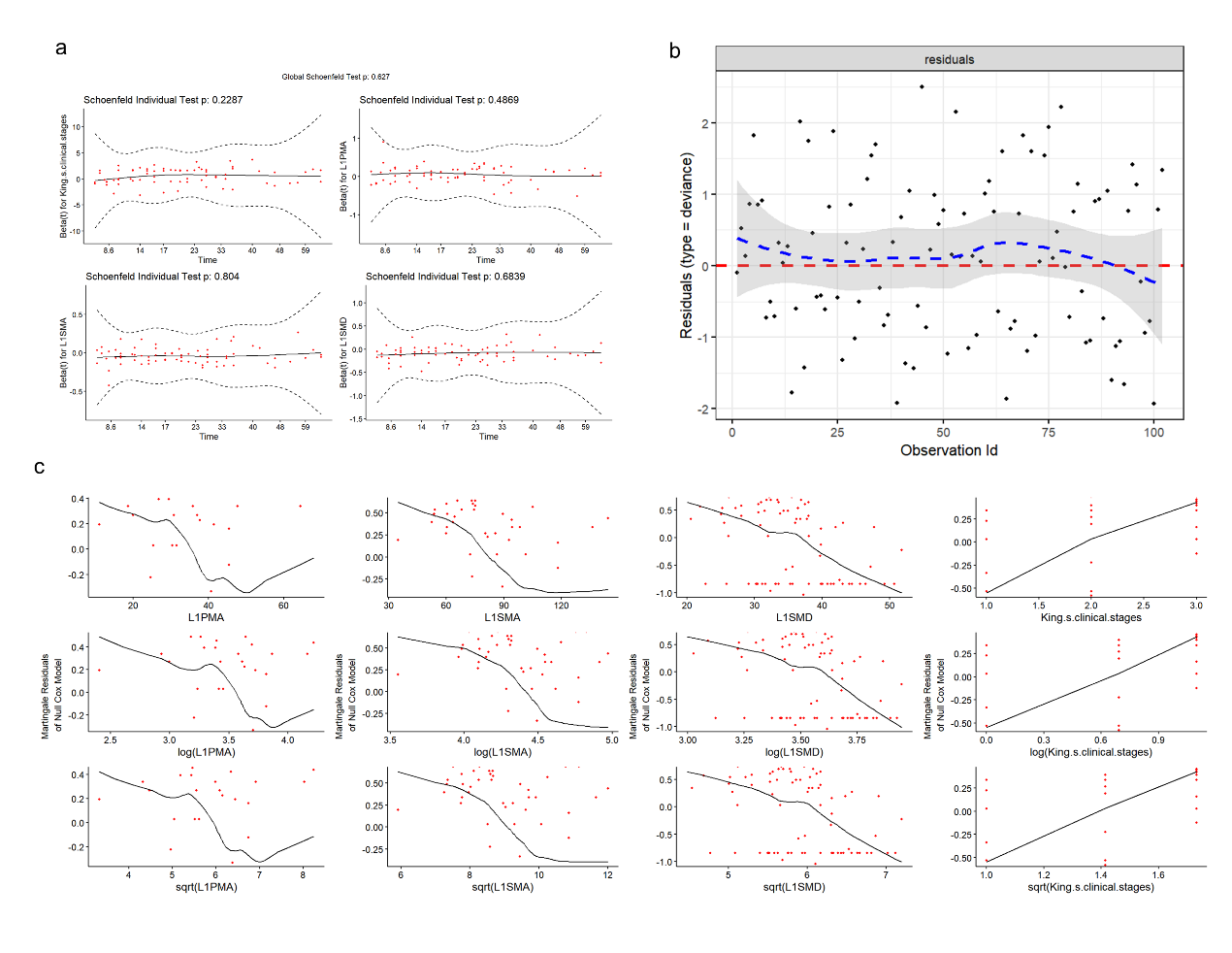


**Figure S4. Cox proportional risk regression models for L1-SMA, L1-PMD, and King’s clinical stages.** (a) Standardized (scaled) Schoenfeld residuals are independent of survival time, indicating the proportional risk assumption is satisfied; (b) Deviance residuals are dispersed and symmetric; (c) Martingale residual plots and partial residual plots are approximately linear.


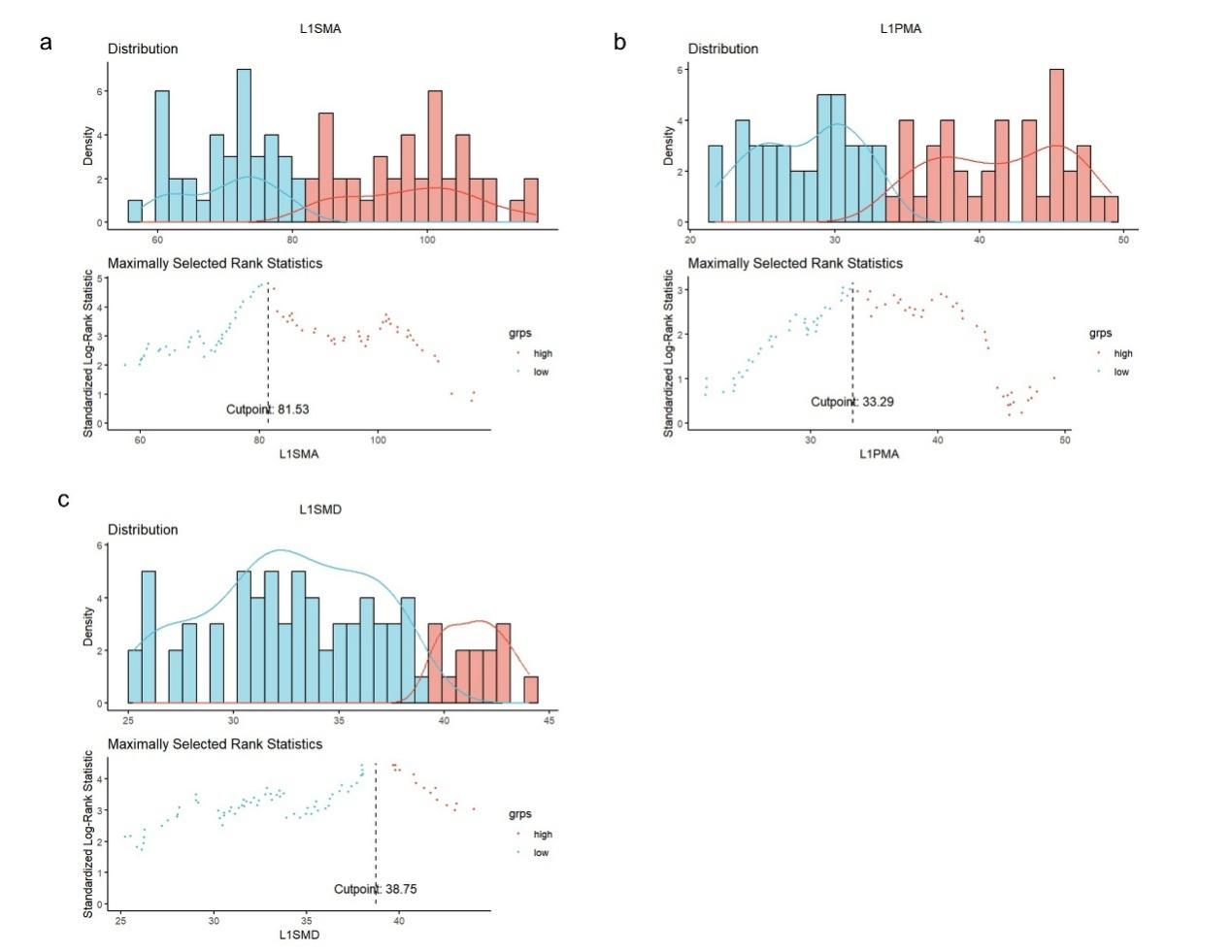


**Figure S5. The maximum-choice log-rank test was used to determine thresholds for quantitative CT metrics**, identifying the optimal thresholds for survival: (a) L1 SMA was 81.53 cm²; (b) L1 SMD was 38.75 cm²; and (c) L1 PMA was 33.29 HU. L1, First Lumbar Vertebra; L1 SMA, Skeletal Muscle Area at L1; L1 PMA, Paravertebral Muscle Area at L1; L1 PMD, Paravertebral Muscle Density at L1; HU, Hounsfield Units.


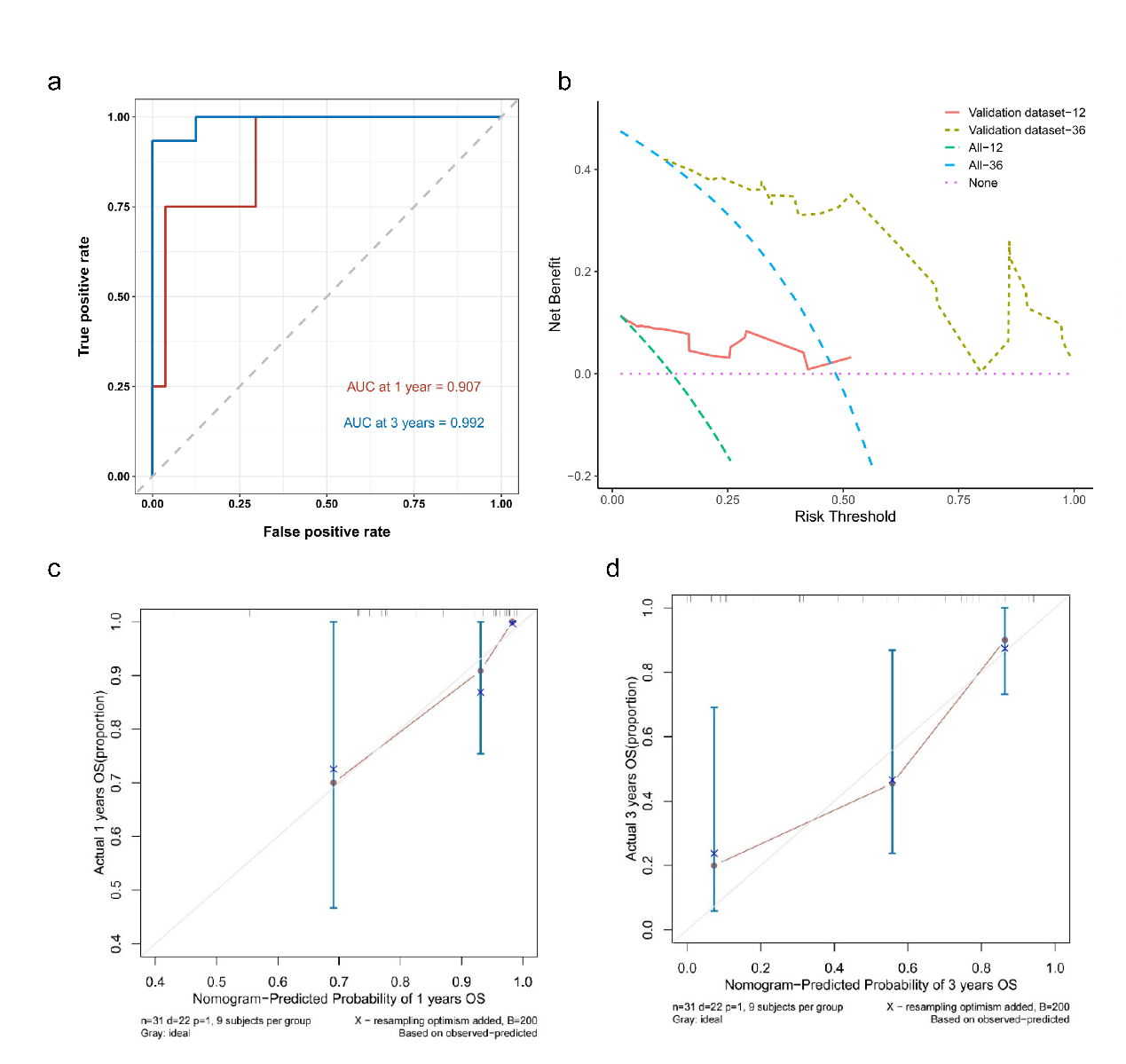


**Figure** **S6. Validation** **set** **cohort:** (a) Receiver Operating Characteristic (ROC) curves of 1 year and 3 years; (b) Decision Curve Analysis (DCA) curves of 12 months and 36 months; Calibration curves of 1 year (c) and 3 years(d). OS, overall survival.


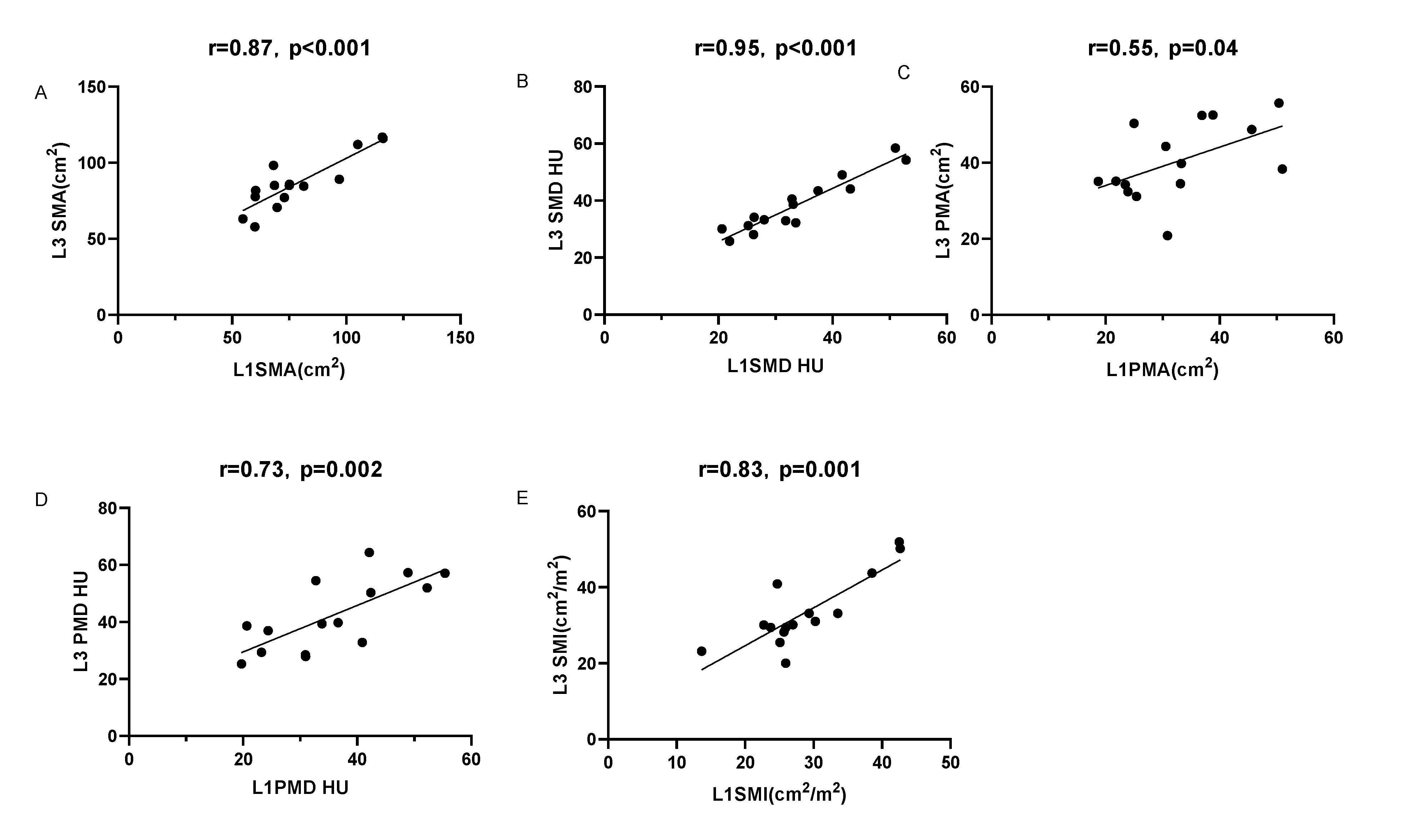


**Figure S7. Association between skeletal muscle parameters at L1 level and L3 level.** L1, The First Lumbar Vertebra; L3=The Third Lumbar Vertebra; SMA= Skeletal Muscle Area; SMD, Skeletal Muscle Density; SMI= Skeletal Muscle Index; PMA, Paravertebral Muscle Area; PMD, Paravertebral Muscle Density; Spearman Correlation.
